# Supplementary material for: Alternatives in Education—Rat and Mouse Simulators Evaluated from Course Trainers’ and Supervisors’ Perspective
Source: Animals (Basel). 2021 Jun 22;11(7):1848. doi: 10.3390/ani11071848 (PMC8300107; doi:10.3390/ani11071848)
Supplement: Supplementary file 1 [file animals-11-01848-s001.zip › Supplementary material_proofedmerged_21.06.21.pdf]

Table S2. Descriptive analysis of Laboratory Animal Science training courses included in the survey. Replies concerning course frequency and number of participants, instructors (trainers and supervisors), as well as animals used in respect to different types of LAS courses performed by the respondents (n = 35). Course types include courses for people carrying out animal experiments (traditionally FELASA B category) and for people planning animal experiments (traditionally FELASA C category). Other courses include courses for humane killing, modular courses or courses for specific procedures. Twenty-four respondents stated that they were course trainers, eight supervisors, and one was both. Two respondents omitted the question.

**General information about course offer**		**Course type**		
		**FELASA B**	**FELASA C**	**Other courses**
Course type	Number of responses[1]	27	10	16
Course frequency per year[2]	Median<br>(Min – Max)	4.00<br>(1.00 – 30.00)	2.00<br>(1.00 – 3.00)	20.00<br>(2.00 – 50.00)
Most frequently performed type of course per year	Number of responses[3]	19 + 2[4]	2[4]	13
Average number of participants per course[5]	Median<br>(Min – Max)	20.00<br>(6.00 – 70.00)	17.00<br>(9.00 – 25.00)	7.00<br>(4.00 – 60.00)
Average number of instructors per course[5]	Median<br>(Min – Max)	4.00<br>(2.00 – 20.00)	3.50<br>(2.00 – 5.00)	2.00<br>(1.00 – 8.00)
Average number of rats per participant in practical training[6]	Median<br>(Min – Max)	1.00<br>(0.50 – 4.00)	0.75<br>(0.50 – 1.00)	1.00<br>(0.50 – 4.00)
Average number of mice per participant in practical training[7]	Median<br>(Min – Max)	2.00<br>(1.00 – 6.00)	1.50<br>(1.00 – 2.00)	3.00<br>(0.50 – 7.00)

[1] Answer frequencies derived from a multiple choice question.

[2] Average number of courses held per year entered by respondents in an numerical input. Course frequencies indicated as higher than 52 times per year were excluded.

[3] Answer frequencies derived from descriptive statistical analysis.

[4] Number of responses indicating equal frequencies in the performance of courses according to traditionally FELASA B and C.

[5] Courses with an instructor per participants ratio below 0.125 (1:8) were excluded.

[6] Courses with an average number of > 10 rats per participant in practical training were excluded.

[7] Courses with an average number of > 20 mice per participant in practical training were excluded.

Table S3. Further methodical and practical requirements stated by the respondents in an open field question. List of comments translated from German language, separated in further methodical and further practical requirements regarding the development of a novel simulator (n = 35).

**Further methodical requirements**
Realistic design of the anatomy (organ size, access to the organ)
Simulators should be possible adapted to the course content ... A "beast of burden" is not to be recommended. Ideally, basic models for different criteria should be available ... E.g. natural replica of the tail veins and arteries for practicing the blood collection technique.
Penetration of the skin must be close to reality. Penetration must correspond to the anatomical conditions. Feedback mechanism (did the method work?)
haptics
Feasibility of the aforementioned techniques in an approximately realistic manner
"true to life"
Must be easy for participants to use
versatile
I would especially like to see a realistic simulator for cervical dislocation, as well as oral gavage
**Further practical requirements**
easy to manipulate, spare parts (replace)
resilient, understandable
The simulator, unlike cadavers, should simulate defensive movements
Costs must remain within limits, also for spare parts Wear should be as low as possible Spare parts should be available at low cost
Especially blood vessels are important in this context (especially the tail vein would be advantageous).<br>The more similar the "skin" and underlying tissues are, the more realistically handling and restraint can be practiced. In contrast, the larynx does not play a role, as the swallowing reflex for oral application cannot be simulated realistically
The user wants to take away a sense of achievement. That is, possibly think about different degrees of severity of the model interventions (one with larger vessels than in nature and then some with true-to-nature size ratios).<br>easy to clean and disinfect
Size and feel should be very similar to the real animal; no openings that anyone can just slide into, but realistic recreation of the conditions
I think the use of simulators is very limited. Students have the most problems when dealing with awake, struggling and vocal mice and rats. They have inhibitions about touching and restraining the animals correctly or lose control when an animal moves. Placing a cannula correctly or making a correct incision is relatively easy in comparison.
"true to life"
because we have many participants at the same time and organize many courses, it has to be very practical to use (storage, cleaning, ...)
easy handling

Table S4. Feedback messages stated by the respondents in a text field, translated from German language.

**List of messages translated from German language stated by the respondents in a text field at the end of the questionnaire (n = 35).**
Thank you for the opportunity to participate in the survey, which will hopefully lead to a practical new development. A nice project, have fun with it!
I think your idea is absolutely great, to create an animal model, an animal simulator that stands out from all the models commercially available on the market and meets the demands of humans, animals and science alike. I hope you are successful and I am looking forward to using such a simulator in our courses in the near future.
The estimated time for filling out the questionnaire seems to me to be clearly understated. A realistic indication would promote confidence.
It is good that a new simulator is being developed based on user needs. In my opinion, it would be good if there were a modular structure, i.e. the simulator could be individually adapted. Since it is planned to produce the simulator via 3D printing, this might be a possible option. This would perhaps also allow the price aspect to be adjusted, as the funds for investments that do not relate to the project are always very limited, especially at research institutions.
Great if this survey leads to simulators being developed further
As already indicated above, I consider the use of simulators to be of limited value, as the real challenge in the courses lies in the correct handling and (for applications) in the correct restraint of the animals. We have quite a large mouse husbandry with many genetically modified mice, where a three-digit number of mice have to be killed every day: many of them due to the wrong sex or genotype. Accordingly, we can easily provide several dead mice for exercises for all course participants without having to breed animals for the course. Dead mice are a much better model than a simulator for many applications. We use them for dissection (anatomy), practising injection techniques, practising blood sampling (works well on freshly killed animal), practising shaving/cutting/sewing/clamping, practising vasectomy, practising transcardiac perfusion, practising cervical dislocation. I see the most potential for replacing dead mice with a simulator in shaving/cutting/sewing/clamping. The knot-only technique can be practised well on homemade suture models. For cutting/sewing/clamping/gluing we had already used chicken wings. I have not yet come across a simulator that correctly simulates bleeding (intentional/accidental) or the absence of bleeding, but I am open to new developments in this field. It is important to me to note that NO SIMULATOR CAN REPLACE THE EXPERIENCE WITH A WAKE ANIMAL. Anaesthetised animals MUST also remain an integral part of training (transcardiac perfusion on an anaesthetised animal looks very different from that on a dead animal/simulator). Simulators can only replace dead animals to a very limited extent.
It would be great if there were finally good and realistic models that are also affordable. The previous ones are gathering dust somewhere in the cupboard...
It would be nice if there is a development. "Dry runs" are important if participants do not yet know how to handle syringes and, if necessary, forceps and surgical equipment. (Course participants often know how to use the pipette).

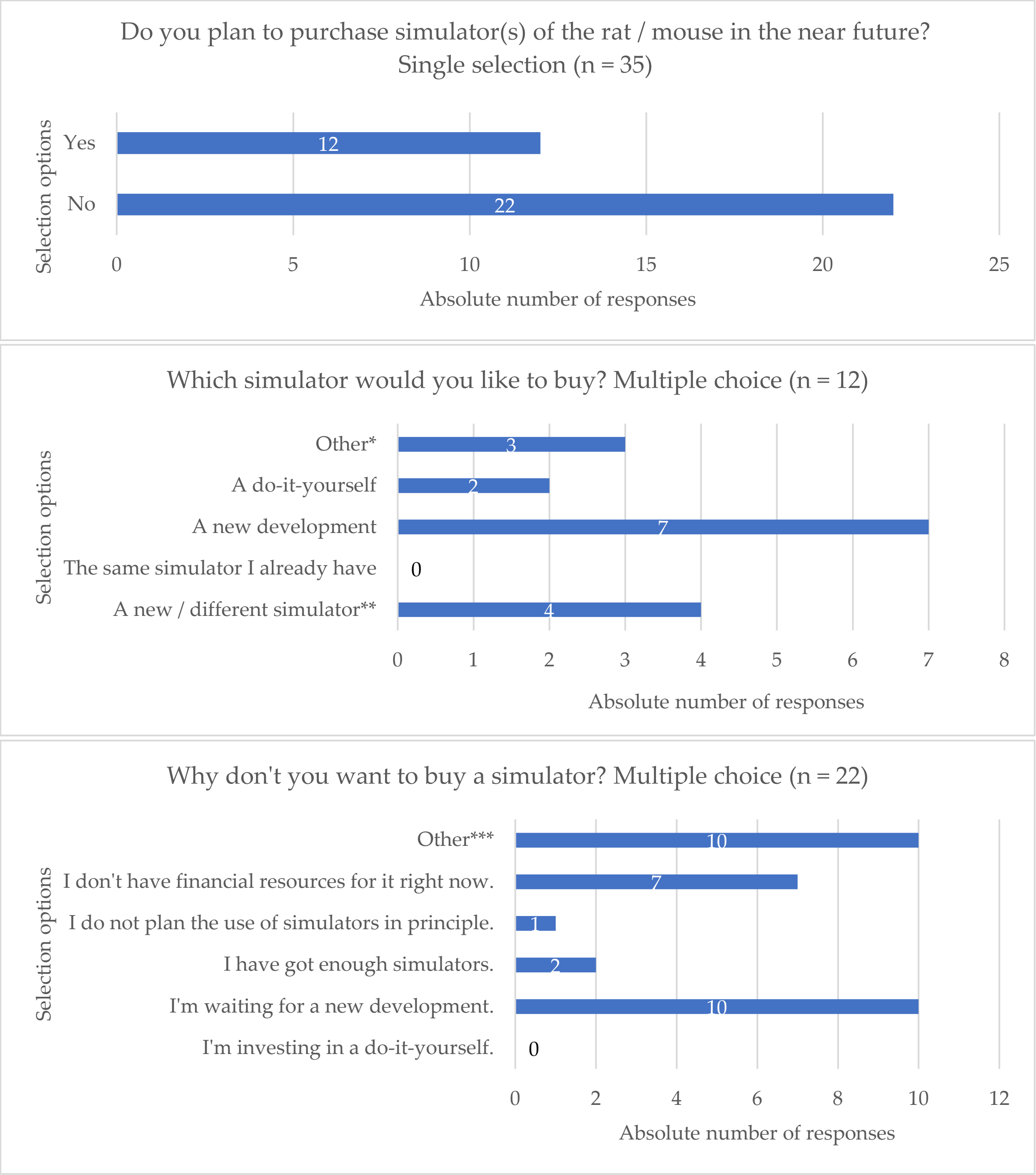

---

* Comments given: Not yet decided/unclear (n = 3)
** Comments given: Mouse simulator (n = 3); rat simulator F (n = 1)
*** Comments given: Inefficient use of simulators (n = 5); no application of simulators planned so far (n = 4); costs (n = 2); Other reasons (n = 1)

Figure S1. Descriptive analysis of the general demand for simulators
First bar chart is showing replies concerning the demand for a novel simulator in terms of a "yes/no" question (n = 35 in total). In case of approval (n = 12), the distribution of replies concerning type of simulator to be purchased in the near future is shown in second bar chart derived from a multiple choice question. In case of rejection (n = 22), the distribution of replies concerning reasons for rejection is shown in the third bar chart derived from a multiple choice question. Response suggestions are listed on the y-axis, response frequencies are shown on the x-axis. Absolute number of answers for each option is given in the associated bar.

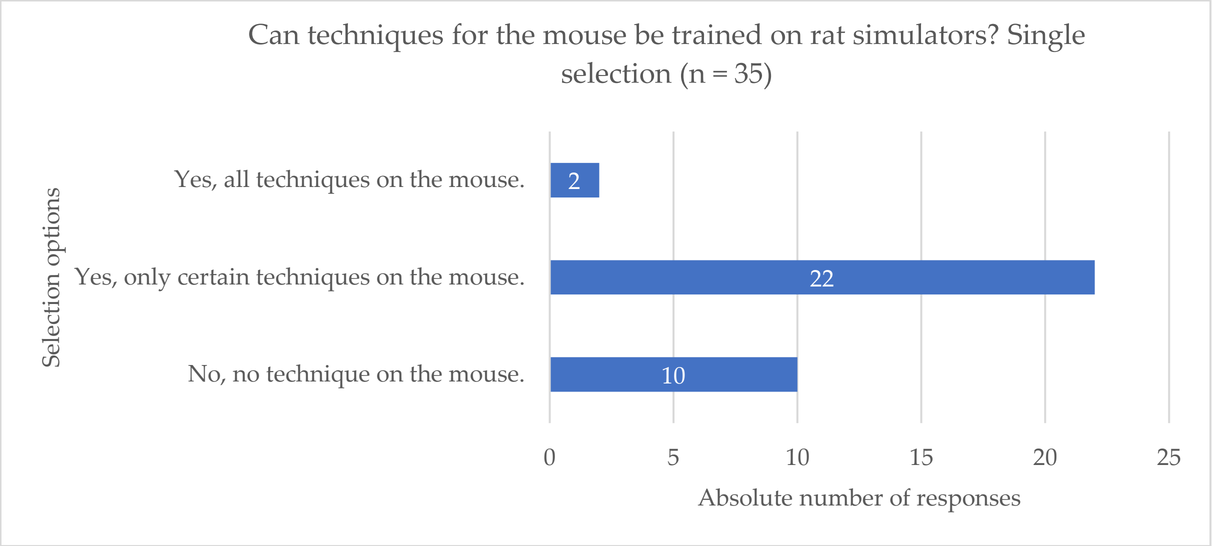

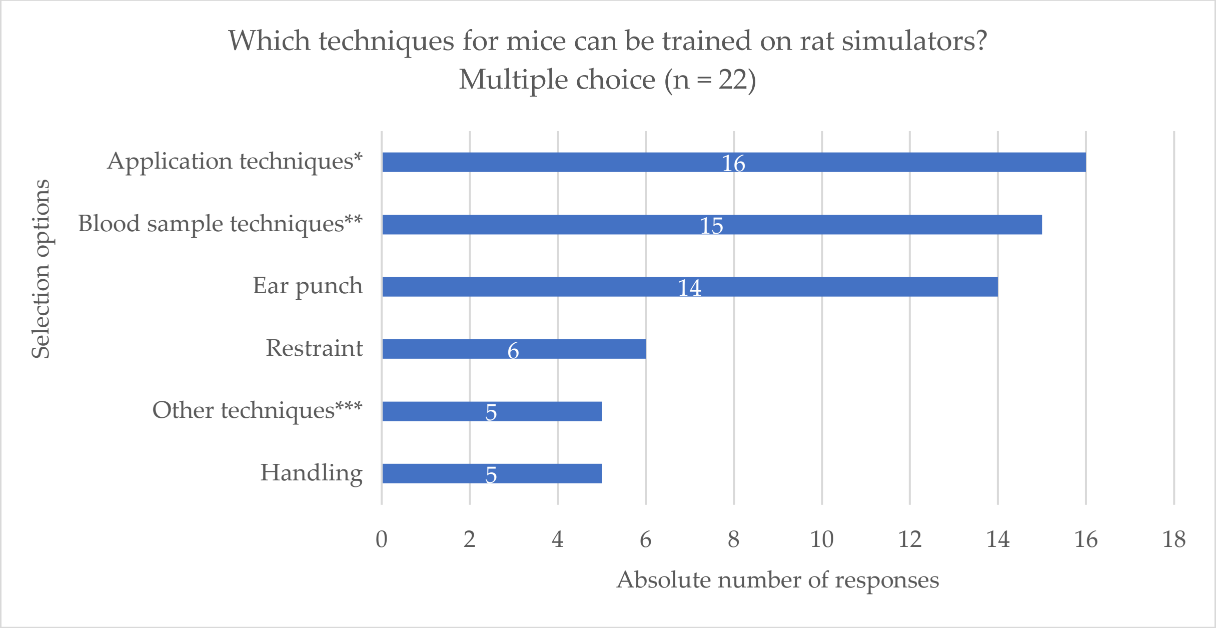

* Subcutaneous (n = 15), intraperitoneal (n = 13), intramuscular (n = 8), and intravenous (n = 6) administration

** Blood sampling via tail vein (n = 10), heart (n = 8), saphenous vein (n = 7), retro-bulbar plexus (n = 4), and facial vein (n = 4)

*** Cycle control (n = 1), cervical dislocation (n = 1), microsurgical (n = 1) and suture techniques (n = 1)

Figure S2. Interspecies transferability of techniques
Replies on the transferability of techniques from rat simulator to mouse. First bar chart is showing replies concerning the feasibility of training mouse techniques on rat simulators derived from a single selection question (n = 35). Second bar chart is showing replies concerning the types of mouse techniques trainable on rat simulators derived from a multiple choice question (n = 22). Response suggestions are listed on the y-axis, response frequencies are shown on the x-axis. Absolute number of answers for each option is given in the associated bar.

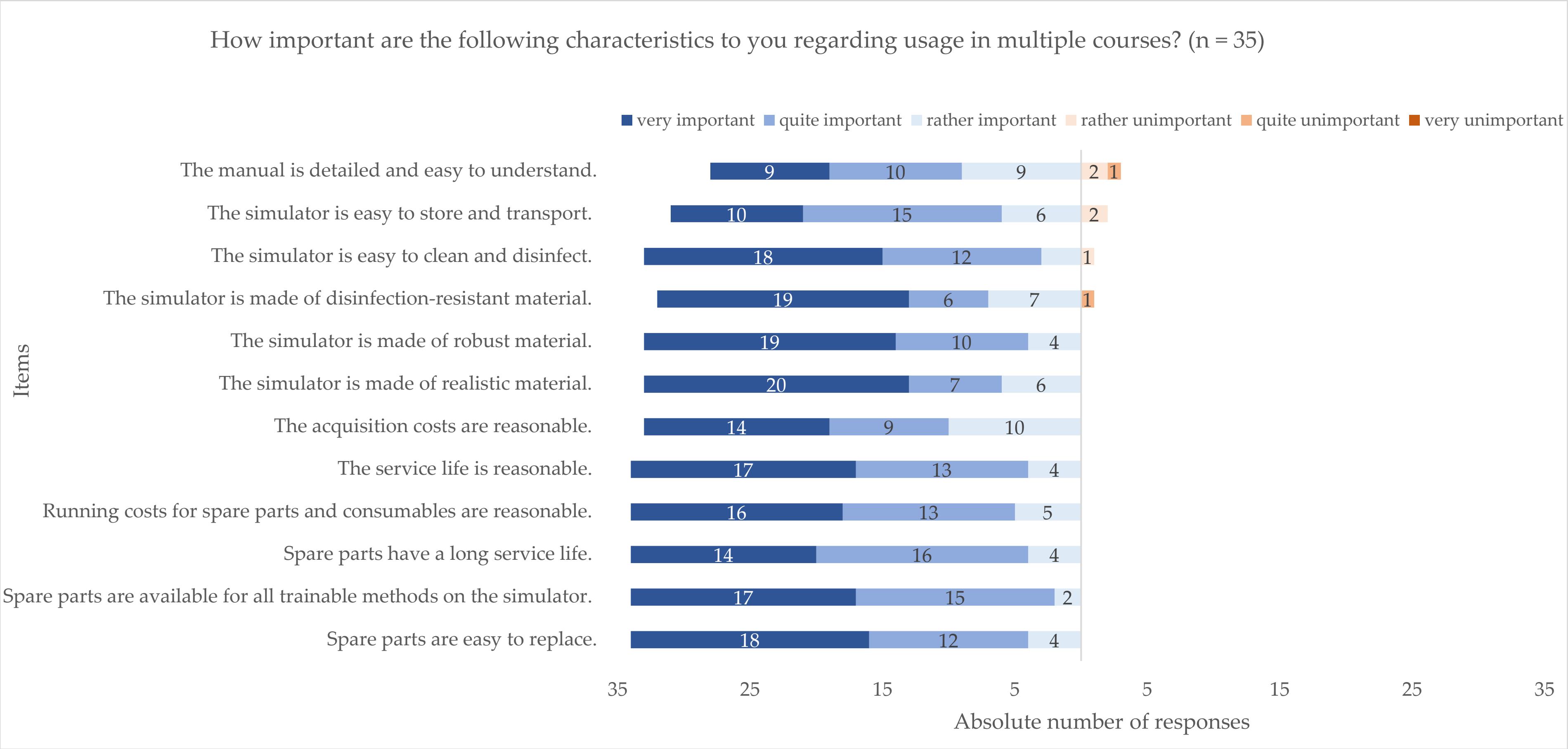

Figure S3. Descriptive analysis of practical requirements for a novel simulator

Distribution of replies concerning the importance of certain practical characteristics on a six-point Likert scale, referring to multiple usage in future courses. Data are shown in a diverging stacked bar chart. Statements about practicability are listed on the y-axis, response frequencies on the x-axis, ranging from “very important” (dark blue), “quite important” (blue), “rather important” (light blue) on left- to “rather unimportant” (light red), “quite unimportant” (red), “very unimportant” (dark red) on the right-handed diverging stacked bars. Absolute number of responses is shown for item in the corresponding bar (n = 35).
