# Supplementary material for: Alternatives in Education—Rat and Mouse Simulators Evaluated from Course Trainers’ and Supervisors’ Perspective
Source: Animals (Basel). 2021 Jun 22;11(7):1848. doi: 10.3390/ani11071848 (PMC8300107; doi:10.3390/ani11071848)
Supplement: Supplementary file 1 [file animals-11-01848-s001.zip › Table S1_proofedmerged_21.06.21.pdf]

Table S1. Questionnaire for LAS course trainers and supervisors

PDF file of the original German questionnaire exported from the free and open source online survey web app "Limesurvey" [28] (page 1 - 48) and translated version of the PDF file (page 49 - 96). The questionaire was conducted online from 31 March 2018 until 30 June 2019 using "Limesurvey" [28]. Simulator product names were anonymized by coding (Rat simulator B, A, C, D, E, Mouse simulator and Rat simulator F) and illustrations were subsequently omitted in the PDF file. For illustrations of the simulators, see Table 1.

References

28. Schmitz, C. Startseite - LimeSurvey - einfache Online-Umfragen. Available online: https://www.limesurvey.org/de/ (accessed on 30 March 2021).

Teil A: Sehr geehrte Kursleiter, sehr geehrte Kursleiterinnen, sehr geehrte Kurs-

betreuer, sehr geehrte Kursbetreuerinnen,

in dem Bf3R-geförderten Kooperationsprojekt „SimulRATor" am Fachbereich Veterinärmedizin der Freien Universität Berlin entwickeln wir, ein Team aus Mitarbeitern der Institute für Veterinär-Anatomie, für Tierschutz, Tierverhalten und Versuchstierkunde und für Veterinär-Epidemiologie und Biometrie einen anatomisch korrekten und kostengünstigen Simulator für versuchstierkundliche Kurse.

Um den Simulator optimal auf den Kurseinsatz auszurichten, evaluieren wir im Projekt „SimulRATor" die derzeit erhältlichen Ratten- und Maussimulatoren aus Sicht der Kursleiter/innen (inklusive Kursbetreuer/innen) und der Kursteilnehmer/innen sowie nach Maßgabe der derzeit gültigen Tierschutzgesetzregelungen. In zwei anonymen online-Umfragen werden die Nutzung und die Zufriedenheit über Simulatoren erfasst, sowie Ansprüche und Bedürfnisse an einen neuen Simulator aufgestellt.

Die aktuelle Umfrage richtet sich an Alle, die versuchstierkundliche Kurse für Ratte und Maus durchführen - mit oder ohne Anwendung von Simulatoren und dient der Beurteilung von Simulatoren aus Sicht der Kursleiter/innen und Kursbetreuer/innen.

Alle Ergebnisse fließen in eine abschließende Anforderungsanalyse, auf deren Grundlage am Institut für Veterinär-Anatomie ein neuer Simulator mittels 3D-Druck erstellt wird.

Bitte nehmen Sie sich ca. 15 Minuten Zeit. Ihre Erfahrungen und Ihr Engagement helfen, einen Simulator zu entwickeln, der die Ansprüche von Mensch, Tier und Wissenschaft gleichermaßen berücksichtigt.

Hinweis zum Datenschutz: Ihre Teilnahme an der Umfrage ist freiwillig. Die Umfragedaten werden anonymisiert gespeichert und verwaltet. Es sind keine Rückschlüsse auf Ihre Teilnahme möglich. Alle Daten werden unter strengster Berücksichtigung der aktuellen Datenschutzrichtlinien verwaltet. Nur Projektbeteiligte haben Zugriff auf die Daten und stehen unter Schweigepflicht. Keinesfalls werden Daten an Dritte weitergegeben. Die Daten werden ausschließlich für Forschungszwecke genutzt. Sie können die Umfrage jederzeit ohne Angabe von Gründen abbrechen und Ihre Daten löschen, in dem Sie das Browserfenster schließen.

Wir bedanken uns herzlichst für Ihre Teilnahme!

Teil B: Allgemeines

Bitte benutzen Sie die Schaltflächen "Zurück" und "Weiter" um zurück- oder vorwärts zu gehen.
Die Umfrage kann jederzeit unter Später fortfahren zwischengespeichert werden. Ihre zwischengespeicherte Umfrage können Sie auf der Startseite laden. Zwischenspeichern empfiehlt sich für Unterbrechungen von mehr als 30 Minuten.

Bitte beantworten Sie alle folgenden Fragen für den Kurs, den Sie am häufigsten durchführen.

B1. Welche Kurse für Ratte und/oder Maus führen Sie durch?

- Kurs für Personen, die Tierversuche durchführen (ehemalig FELASA Kategorie B-Kurs) ☐
- Kurs für Personen, die Tierversuche planen (ehemalig FELASA Kategorie C-Kurs) ☐
- sonstige Kurse ☐
- keine ☐

B2. Wie viele Kurse führen Sie pro Jahr durch? Bitte geben Sie nur Zahlen ein.

- Kurs für Personen, die Tierversuche durchführen (ehemalig FELASA Kategorie B-Kurs) ☐☐☐☐
- Kurs für Personen, die Tierversuche planen (ehemalig FELASA Kategorie C-Kurs) ☐☐☐☐
- sonstige Kurse ☐☐☐☐

B3. Aus wie vielen teilnehmenden und kursbetreuenden Personen besteht Ihr Kurs im Durchschnitt?

Bitte geben Sie nur Zahlen ein.

- Anzahl Teilnehmer/innen ☐☐☐☐☐
- Anzahl kursbetreuender Personen (Kursleiter/innen & Kursbetreuer/innen) ☐☐☐☐☐

Teil C: Ergänzende Methoden

C1. Welche Methoden setzen Sie als Ergänzung im Kurs ein?

- Anleitungen, Zeichnungen, Fotos ☐
- Videos ☐
- Lernsoftware (on-/offline) ☐
- Computersimulationen ☐
- Stofftiere/Puppen ☐
- Simulatoren für die Ratte ☐
- Simulatoren für die Maus ☐

Nahtpads ☐

Sonstige/Selbstbau ☐

keine ☐

C2. Welche sonstigen Methoden setzen Sie ein? Bedingung C1: Auswahl "sonstige/Selbstbau".

Bitte beschreiben Sie diese kurz.

C3. Wie zufrieden sind Sie mit Ihren ergänzenden Methoden?

	sehr zufrieden	ziemlich zufrieden	eher zufrieden	eher unzufrieden	ziemlich unzufrieden	sehr unzufrieden	keine Angabe
Anleitungen, Zeichnungen, Fotos	☐	☐	☐	☐	☐	☐	☐
Videos	☐	☐	☐	☐	☐	☐	☐
Lernsoftware (on-/offline)	☐	☐	☐	☐	☐	☐	☐
Computersimulationen	☐	☐	☐	☐	☐	☐	☐
Stofftiere/Puppen	☐	☐	☐	☐	☐	☐	☐
Simulatoren für die Ratte	☐	☐	☐	☐	☐	☐	☐
Simulatoren für die Maus	☐	☐	☐	☐	☐	☐	☐
Nahtpads	☐	☐	☐	☐	☐	☐	☐
Sonstige/Selbstbau	☐	☐	☐	☐	☐	☐	☐
keine	☐	☐	☐	☐	☐	☐	☐

Teil D (Bedingung: C1 Auswahl Nahtpads): Ergänzung durch Nahtpads

D1. Wie viele Nahtpads setzen Sie im Kurs ein?

Nahtpads ☐☐☐☐ Stk.

D2. Seit wann werden Nahtpads in Ihrem Kurs eingesetzt?

seit etwa ☐☐☐ Jahren

Teil E: Simulatoren der Ratte und Maus

E1. Welche Simulatoren für Ratte und Maus sind Ihnen bekannt? Jeweils 1 Antwort ist möglich.

	nicht bekannt	theoretisch bekannt	sporadischer Kurseinsatz	regelmäßiger Kurseinsatz
Rat simulator B	☐	☐	☐	☐
Rat simulator A	☐	☐	☐	☐
Rat simulator C	☐	☐	☐	☐
Rat simulator D	☐	☐	☐	☐
Rat simulator E	☐	☐	☐	☐
Mouse simulator	☐	☐	☐	☐
Rat simulator F	☐	☐	☐	☐

E2. (Bedingung E1 Auswahl "sporadische Nutzung" oder "regelmäßige Nutzung" > 0)

Wie häufig arbeiten Sie mit dem/den ausgewählten Simulator/en?

	nie	in ca. 1/4 aller Kurse	in ca. 1/2 aller Kurse	in ca. 3/4 aller Kurse	in allen Kursen	andere Häufigkeit
Rat simulator B	☐	☐	☐	☐	☐	☐
Rat simulator A	☐	☐	☐	☐	☐	☐
Rat simulator C	☐	☐	☐	☐	☐	☐
Rat simulator D	☐	☐	☐	☐	☐	☐
Rat simulator E	☐	☐	☐	☐	☐	☐
Mouse simulator	☐	☐	☐	☐	☐	☐
Rat simulator F	☐	☐	☐	☐	☐	☐

E3. **(Bedingung E1 Auswahl entsprechender Simulator)**
In welchem Häufigkeitsverhältnis arbeiten Sie mit dem/den ausgewählten Simulator/en?

Bitte geben Sie die Antwort im Verhältnis aller Kurse an oder nutzen Sie das Feld für eine Beschreibung.

Ratsimulator B

Rat simulator A

Rat simulator C

Rat simulator D

Rat simulator E

Mouse simulator

Rat simulator F

E4. **(Bedingung E1 Auswahl entsprechender Simulator)**
Wie viele Exemplare setzen Sie jeweils im Kurs ein?

Bitte geben Sie nur Zahlen ein.

Rat simulator B

Rat simulator A

Rat simulator C

Rat simulator D

Rat simulator E

Mouse simulator

Rat simulator F

E5. **(E1 Auswahl "sporadische Nutzung" oder "regelmäßige Nutzung" entsprechender Simulator > 1)**

Mit welchem Ihrer Simulatoren arbeiten Sie bevorzugt?

- [ ] Rat simulator B
- [ ] Rat simulator A
- [ ] Rat simulator C
- [ ] Rat simulator D
- [ ] Rat simulator E
- [ ] Mouse simulator
- [ ] Rat simulator F
- [ ] keiner
- [ ] alle gleich

Teil F: (E1 Auswahl "sporadische Nutzung" oder "regelmäßige Nutzung" entsprechender Simulator > 0)

Ergänzung durch Simulatoren

F1. Wie nutzen Sie den/die ausgewählten Simulator/en im praktischen Kursteil?

Mehrere Antworten sind möglich.

- Demonstration der Techniken durch betreuende Personen inkl. Kursleiter/innen ☐
- Training der Teilnehmer/innen am Simulator in einer separaten Kurseinheit ☐
- Training der Teilnehmer/innen am Simulator parallel zur Übung am Tier ☐
- sonstiges ☐

F2. Trainieren alle Kursteilnehmer/innen an den Simulatoren oder ist das Training für einzelne Kursteilnehmer/innen bestimmt?

- Ja, das Training ist verpflichtend. ☐
- Ja, das Training ist freiwillig. ☐
- Nein, das Training ist für einzelne Kursteilnehmer/innen bestimmt. ☐
- Sonstiges ☐

Sonstiges

[____________________]

F3. Planen Sie in nächster Zeit Simulatoren für Ratte und/oder Maus anzuschaffen?

- Ja ☐
- Nein ☐

F4. Welchen Simulator möchten Sie sich anschaffen?

Mehrere Antworten sind möglich.

- den gleichen Simulator, den ich schon habe: ☐
- Rat simulator B ☐
- Rat simulator A ☐
- Rat simulator C ☐
- Rat simulator D ☐
- Rat simulator E ☐
- Mouse simulator ☐

Rat simulator F ☐

einen neuen/anderen Simulator: ☐

einen neuen/anderen Simulator: Rat Simulator B ☐

einen neuen/anderen Simulator: Rat Simulator A ☐

einen neuen/anderen Simulator: Rat Simulator C ☐

einen neuen/anderen Simulator: Rat Simulator D ☐

einen neuen/anderen Simulator: Rat Simulator E ☐

einen neuen/anderen Simulator: Mouse Simulator ☐

einen neuen/anderen Simulator: Rat Simulator F ☐

eine Neueentwicklung ☐

einen Selbstbau ☐

sonstiges ☐

sonstiges

F5. Warum möchten Sie sich keinen Simulator anschaffen?

Mehrere Antworten sind ggf. möglich.

Ich plane den Einsatz von Simulatoren grundsätzlich nicht. ☐

Ich habe derzeit keine Mittel dafür. ☐

Ich habe genügend Simulatoren. ☐

Ich warte auf eine Neuentwicklung. ☐

Ich investiere in einen Selbstbau. ☐

sonstiges ☐

sonstiges

Teil G: Übertragbarkeit Ratte → Maus

G1. Können Techniken für die Maus an Rattensimulatoren trainiert werden?

Ja, alle Techniken an der Maus ☐

Ja, nur bestimmte Techniken an der Maus ☐

Nein, keine Technik an der Maus ☐

G2. Welche Techniken für die Maus können an Rattensimulatoren trainiert werden?

- Handling ☐
- Fixation ☐
- Ohrlochmarkierung ☐
- Applikationstechniken ☐
- Injektion subkutan ☐
- Injektion intramuskulär ☐
- Injektion intraperitoneal ☐
- Injektion intravenös Vena caudalis mediana ☐
- Blutentnahmetechniken ☐
- Vena facialis ☐
- retrobulbärer Venenplexus ☐
- Vena saphena ☐
- Vena caudalis mediana ☐
- intrakardial ☐
- sonstige Techniken ☐
- Vaginalabstrich ☐
- zervikale Dislokation ☐
- mikrochirurgische Techniken ☐
- Nahttechnik ☐
- Sonstiges ☐

G3. Welche sonstigen Techniken für die Maus können an Rattensimulatoren trainiert werden?

Teil H: Die Übung am Tier

H1. Welche Methoden an Ratte und/oder Maus vermitteln Sie im praktischen Kursteil? Bitte geben Sie an, ob die Übung an der wachen, narkotisierten und/oder toten Ratte bzw. Maus durchgeführt wird.

Mehrere Antworten sind möglich.

SQ001:

	Ratte				Maus			
	wach	narkotisiert	Post mortem	Kein Training	wach	narkotisiert	Post mortem	Kein Training
Handling	☐	☐	☐	☐	☐	☐	☐	☐
Fixation	☐	☐	☐	☐	☐	☐	☐	☐
Ohrlochmarkierung	☐	☐	☐	☐	☐	☐	☐	☐
Applikation per os mittels Sonde	☐	☐	☐	☐	☐	☐	☐	☐
Applikation per os ohne Sonde	☐	☐	☐	☐	☐	☐	☐	☐
Injektion subkutan	☐	☐	☐	☐	☐	☐	☐	☐
Injektion intramuskulär	☐	☐	☐	☐	☐	☐	☐	☐
Injektion intraperitoneal	☐	☐	☐	☐	☐	☐	☐	☐
Injektion intravenös dorsale Penisvene (Ratte)	☐	☐	☐	☐	☐	☐	☐	☐
Injektion intravenös Vena caudalis mediana	☐	☐	☐	☐	☐	☐	☐	☐
Blutentnahme Vena sublingualis (Ratte)	☐	☐	☐	☐	☐	☐	☐	☐
Blutentnahme Vena facialis (Maus)	☐	☐	☐	☐	☐	☐	☐	☐
Blutentnahme retrobulbärer Plexus	☐	☐	☐	☐	☐	☐	☐	☐
Blutentnahme Vena saphena	☐	☐	☐	☐	☐	☐	☐	☐
Blutentnahme Vena caudalis mediana	☐	☐	☐	☐	☐	☐	☐	☐
Blutentnahme intrakardial	☐	☐	☐	☐	☐	☐	☐	☐
Zervikale Dislokation (bis 100g)	☐	☐	☐	☐	☐	☐	☐	☐
Intubation	☐	☐	☐	☐	☐	☐	☐	☐
Vaginalabstrich	☐	☐	☐	☐	☐	☐	☐	☐
Mikrochirurgische Techniken	☐	☐	☐	☐	☐	☐	☐	☐
Nahttechniken	☐	☐	☐	☐	☐	☐	☐	☐
Sonstiges	☐	☐	☐	☐	☐	☐	☐	☐

H2.b (Bedingung H1: Auswahl "Sonstiges")
Welche sonstigen Methoden werden im praktischen Kursteil vermittelt?

Bitte beschreiben Sie diese kurz.

Ratte wach [ ]

Ratte narkotisiert [ ]

Ratte post mortem [ ]

Maus wach [ ]

Maus narkotisiert [ ]

Maus post mortem [ ]

H3. An wie vielen lebenden Tieren trainieren die Kursteilnehmer/innen im Kurs?

Bitte geben Sie das Verhältnis Tiere zu Kursteilnehmer/innen an.

Anzahl Tiere:

Übung an der Ratte [ ]

Übung an der Maus [ ]

/ Kursteilnehmer/innen:

Übung an der Ratte [ ]

Übung an der Maus [ ]

Teil I: Rat simulator B (Bedingung E1 Auswahl "sporadische Nutzung" oder "regelmäßige Nutzung" für Rat simulator B)

I1. Seit wie vielen Jahren wird Rat simulator B in Ihrem Kurs eingesetzt?

Bitte geben Sie nur Zahlen ein.

seit etwa [ ] Jahren

I2. Wie zufrieden sind Sie mit Rat simulator B insgesamt?

- sehr zufrieden ☐
- ziemlich zufrieden ☐
- eher zufrieden ☐
- eher unzufrieden ☐
- ziemlich unzufrieden ☐
- sehr unzufrieden ☐
- keine Angabe ☐

I3. Wie gut können folgende Methoden an Rat simulator B geübt werden?

Bitte vergeben Sie Schulnoten von 1 bis 6:

1: sehr gut; 2: gut; 3: befriedigend; 4: ausreichend; 5: mangelhaft; 6:ungenügend; kein Training: Methode wird nicht am Simulator trainiert.

	1	2	3	4	5	6	kein Training
Handling	☐	☐	☐	☐	☐	☐	☐
Fixation	☐	☐	☐	☐	☐	☐	☐
Ohrlochmarkierung	☐	☐	☐	☐	☐	☐	☐
Applikation per os mittels Sonde	☐	☐	☐	☐	☐	☐	☐
Applikation per os ohne Sonde	☐	☐	☐	☐	☐	☐	☐
Injektion subkutan	☐	☐	☐	☐	☐	☐	☐
Injektion intramuskulär	☐	☐	☐	☐	☐	☐	☐
Injektion intraperitoneal	☐	☐	☐	☐	☐	☐	☐
Injektion intravenös dorsale Penisvene (Ratte)	☐	☐	☐	☐	☐	☐	☐
Injektion intravenös Vena caudalis mediana	☐	☐	☐	☐	☐	☐	☐
Blutentnahme Vena sublingualis (Ratte)	☐	☐	☐	☐	☐	☐	☐
Blutentnahme Vena facialis (Maus)	☐	☐	☐	☐	☐	☐	☐
Blutentnahme retrobulbärer Venenplexus	☐	☐	☐	☐	☐	☐	☐
Blutentnahme Vena saphena	☐	☐	☐	☐	☐	☐	☐
Blutentnahme Vena caudalis mediana	☐	☐	☐	☐	☐	☐	☐
Blutentnahme intracardial	☐	☐	☐	☐	☐	☐	☐
zervikale Dislokation (bis 100g)	☐	☐	☐	☐	☐	☐	☐
Intubation	☐	☐	☐	☐	☐	☐	☐
Vaginalabstrich	☐	☐	☐	☐	☐	☐	☐
mikrochirurgische Techniken	☐	☐	☐	☐	☐	☐	☐
Nahttechniken	☐	☐	☐	☐	☐	☐	☐
sonstige Methoden	☐	☐	☐	☐	☐	☐	☐

I4. **(Bedingung I3: Auswahl "sonstige Methoden") Bitte beschreiben Sie sonstige Methoden kurz.**

I5. **Wie gut sind folgende anatomische Merkmale an Rat simulator B nachgebildet?**

Bitte vergeben Sie Schulnoten von 1 bis 6:

1: sehr gut; 2: gut; 3: befriedigend; 4: ausreichend; 5: mangelhaft; 6: ungenügend.

	1	2	3	4	5	6	keine Angabe
Haptik allgemein	☐	☐	☐	☐	☐	☐	☐
⇒ speziell: Verschieblichkeit und Konsistenz der Haut	☐	☐	☐	☐	☐	☐	☐
Körpergröße	☐	☐	☐	☐	☐	☐	☐
Gewicht	☐	☐	☐	☐	☐	☐	☐
Körperform	☐	☐	☐	☐	☐	☐	☐
Proportionen	☐	☐	☐	☐	☐	☐	☐
Vorhandensein geschlechtsspezifischer Merkmale	☐	☐	☐	☐	☐	☐	☐
Vorhandensein von Blutgefäßen	☐	☐	☐	☐	☐	☐	☐
Lage der Blutgefäße	☐	☐	☐	☐	☐	☐	☐
Verlauf der Blutgefäße	☐	☐	☐	☐	☐	☐	☐
Beschaffenheit der Blutgefäße	☐	☐	☐	☐	☐	☐	☐
Vorhandensein von Gelenken	☐	☐	☐	☐	☐	☐	☐
Beweglichkeit der Gelenke	☐	☐	☐	☐	☐	☐	☐

I6. Wie praktikabel ist Rat simulator B im Einsatz mehrerer Kurse?

Bitte geben Sie an, wie gut folgende Aussagen über Rat simulator B zutreffen.

	trifft voll und ganz zu	trifft weitgehend zu	trifft eher zu	trifft eher nicht zu	trifft weitgehend nicht zu	trifft überhaupt nicht zu	keine Angabe
Die Gebrauchsanweisung ist ausführlich und verständlich.	☐	☐	☐	☐	☐	☐	☐
Den Simulator kann man gut lagern und transportieren.	☐	☐	☐	☐	☐	☐	☐
Den Simulator kann man gut reinigen und desinfizieren.	☐	☐	☐	☐	☐	☐	☐
Der Simulator ist aus desinfektionsbeständigem Material.	☐	☐	☐	☐	☐	☐	☐
Der Simulator ist aus robustem Material.	☐	☐	☐	☐	☐	☐	☐
Der Simulator ist aus realitätsnahem Material.	☐	☐	☐	☐	☐	☐	☐
Die Anschaffungskosten sind angemessen.	☐	☐	☐	☐	☐	☐	☐
Die Lebensdauer ist angemessen.	☐	☐	☐	☐	☐	☐	☐
Laufende Kosten für Ersatzteile und Verbrauchsmaterialien sind angemessen.	☐	☐	☐	☐	☐	☐	☐
Die Ersatzteile haben eine lange Lebensdauer.	☐	☐	☐	☐	☐	☐	☐
Ersatzteile gibt es für alle Methoden, die am Simulator geübt werden.	☐	☐	☐	☐	☐	☐	☐
Die Ersatzteile lassen sich leicht austauschen.	☐	☐	☐	☐	☐	☐	☐

Teil J: Rat simulator A (Bedingung E1 Auswahl "sporadische Nutzung" oder "regelmäßige Nutzung" für Rat simulator A)

J1. Seit wie vielen Jahren wird die Rat simulator A in Ihrem Kurs eingesetzt?

Bitte geben Sie nur Zahlen ein.

seit etwa ☐☐☐☐ Jahren

J2. Wie zufrieden sind Sie mit Rat simulator A insgesamt?

- sehr zufrieden ☐
- ziemlich zufrieden ☐
- eher zufrieden ☐
- eher unzufrieden ☐
- ziemlich unzufrieden ☐
- sehr unzufrieden ☐
- keine Angabe ☐

J3. Wie gut können folgende Methoden an Rat simulator A geübt werden?

Bitte vergeben Sie Schulnoten von 1 bis 6:

1: sehr gut; 2: gut; 3: befriedigend; 4: ausreichend; 5: mangelhaft; 6: ungenügend;

kein Training: Methode wird nicht am Simulator trainiert.

	1	2	3	4	5	6	kein Training
Handling	☐	☐	☐	☐	☐	☐	☐
Fixation	☐	☐	☐	☐	☐	☐	☐
Ohrlochmarkierung	☐	☐	☐	☐	☐	☐	☐
Applikation per os mittels Sonde	☐	☐	☐	☐	☐	☐	☐
Applikation per os ohne Sonde	☐	☐	☐	☐	☐	☐	☐
Injektion subkutan	☐	☐	☐	☐	☐	☐	☐
Injektion intramuskulär	☐	☐	☐	☐	☐	☐	☐
Injektion intraperitoneal	☐	☐	☐	☐	☐	☐	☐
Injektion intravenös dorsale Penisvene (Ratte)	☐	☐	☐	☐	☐	☐	☐
Injektion intravenös Vena caudalis mediana	☐	☐	☐	☐	☐	☐	☐
Blutentnahme Vena sublingualis (Ratte)	☐	☐	☐	☐	☐	☐	☐
Blutentnahme Vena facialis (Maus)	☐	☐	☐	☐	☐	☐	☐
Blutentnahme retrobulbärer Venenplexus	☐	☐	☐	☐	☐	☐	☐
Blutentnahme Vena saphena	☐	☐	☐	☐	☐	☐	☐
Blutentnahme Vena caudalis mediana	☐	☐	☐	☐	☐	☐	☐
Blutentnahme intrakardial	☐	☐	☐	☐	☐	☐	☐
zervikale Dislokation (bis 100g)	☐	☐	☐	☐	☐	☐	☐
Intubation	☐	☐	☐	☐	☐	☐	☐
Vaginalabstrich	☐	☐	☐	☐	☐	☐	☐
mikrochirurgische Techniken	☐	☐	☐	☐	☐	☐	☐
Nahttechniken	☐	☐	☐	☐	☐	☐	☐

J4. (Bedingung J3: Auswahl "sonstige Methoden") Bitte beschreiben Sie sonstige Methoden kurz.

J5. Wie gut sind folgende anatomische Merkmale an Rat simulator A nachgebildet?

Bitte vergeben Sie Schulnoten von 1 bis 6:

1: sehr gut; 2: gut; 3: befriedigend; 4: ausreichend; 5: mangelhaft; 6: ungenügend.

	1	2	3	4	5	6	keine Angabe
Haptik allgemein	☐	☐	☐	☐	☐	☐	☐
⇒ speziell: Verschieblichkeit und Konsistenz der Haut	☐	☐	☐	☐	☐	☐	☐
Körpergröße	☐	☐	☐	☐	☐	☐	☐
Gewicht	☐	☐	☐	☐	☐	☐	☐
Körperform	☐	☐	☐	☐	☐	☐	☐
Proportionen	☐	☐	☐	☐	☐	☐	☐
Vorhandensein geschlechtsspezifischer Merkmale	☐	☐	☐	☐	☐	☐	☐
Vorhandensein von Blutgefäßen	☐	☐	☐	☐	☐	☐	☐
Lage der Blutgefäße	☐	☐	☐	☐	☐	☐	☐
Verlauf der Blutgefäße	☐	☐	☐	☐	☐	☐	☐
Beschaffenheit der Blutgefäße	☐	☐	☐	☐	☐	☐	☐
Vorhandensein von Gelenken	☐	☐	☐	☐	☐	☐	☐
Beweglichkeit der Gelenke	☐	☐	☐	☐	☐	☐	☐

J6. Wie praktikabel ist Rat simulator A im Einsatz mehrerer Kurse?

Bitte geben Sie an, wie gut folgende Aussagen über Rat simulator A zutreffen.

	trifft voll und ganz zu	trifft weitgehend zu	trifft eher zu	trifft eher nicht zu	trifft weitgehend nicht zu	trifft überhaupt nicht zu	keine Angabe
Die Gebrauchsanweisung ist ausführlich und verständlich.	☐	☐	☐	☐	☐	☐	☐
Den Simulator kann man gut lagern und transportieren.	☐	☐	☐	☐	☐	☐	☐
Den Simulator kann man gut reinigen und desinfizieren.	☐	☐	☐	☐	☐	☐	☐
Der Simulator ist aus desinfektionsbeständigem Material.	☐	☐	☐	☐	☐	☐	☐
Der Simulator ist aus robustem Material.	☐	☐	☐	☐	☐	☐	☐
Der Simulator ist aus realitätsnahem Material.	☐	☐	☐	☐	☐	☐	☐
Die Anschaffungskosten sind angemessen.	☐	☐	☐	☐	☐	☐	☐
Die Lebensdauer ist angemessen.	☐	☐	☐	☐	☐	☐	☐
Laufende Kosten für Ersatzteile und Verbrauchsmaterialien sind angemessen.	☐	☐	☐	☐	☐	☐	☐
Die Ersatzteile haben eine lange Lebensdauer.	☐	☐	☐	☐	☐	☐	☐
Ersatzteile gibt es für alle Methoden, die am Simulator geübt werden.	☐	☐	☐	☐	☐	☐	☐
Die Ersatzteile lassen sich leicht austauschen.	☐	☐	☐	☐	☐	☐	☐

Teil K: Rat simulator C (Bedingung E1 Auswahl "sporadische Nutzung" oder "regelmäßige Nutzung" für Rat simulator C)

K1. Seit wie vielen Jahren wird die Rat simulator C in Ihrem Kurs eingesetzt?

Bitte geben Sie nur Zahlen ein.

seit etwa ☐☐☐☐ Jahren

K2. Wie zufrieden sind Sie mit Rat simulator C insgesamt?

- sehr zufrieden ☐
- ziemlich zufrieden ☐
- eher zufrieden ☐
- eher unzufrieden ☐
- ziemlich unzufrieden ☐
- sehr unzufrieden ☐
- keine Angabe ☐

K3. **Wie gut können folgende Methoden an Rat simulator C geübt werden?**

Bitte vergeben Sie Schulnoten von 1 bis 6:

1: sehr gut; 2: gut; 3: befriedigend; 4: ausreichend; 5: mangelhaft; 6: ungenügend; kein Training: Methode wird nicht am Simulator trainiert.

	1	2	3	4	5	6	kein Training
Handling	☐	☐	☐	☐	☐	☐	☐
Fixation	☐	☐	☐	☐	☐	☐	☐
Ohrlochmarkierung	☐	☐	☐	☐	☐	☐	☐
Applikation per os mittels Sonde	☐	☐	☐	☐	☐	☐	☐
Applikation per os ohne Sonde	☐	☐	☐	☐	☐	☐	☐
Injektion subkutan	☐	☐	☐	☐	☐	☐	☐
Injektion intramuskulär	☐	☐	☐	☐	☐	☐	☐
Injektion intraperitoneal	☐	☐	☐	☐	☐	☐	☐
Injektion intravenös dorsale Penisvene (Ratte)	☐	☐	☐	☐	☐	☐	☐
Injektion intravenös Vena caudalis mediana	☐	☐	☐	☐	☐	☐	☐
Blutentnahme Vena sublingualis (Ratte)	☐	☐	☐	☐	☐	☐	☐
Blutentnahme Vena facialis (Maus)	☐	☐	☐	☐	☐	☐	☐
Blutentnahme retrobulbärer Venenplexus	☐	☐	☐	☐	☐	☐	☐
Blutentnahme Vena saphena	☐	☐	☐	☐	☐	☐	☐
Blutentnahme Vena caudalis mediana	☐	☐	☐	☐	☐	☐	☐
Blutentnahme intrakardial	☐	☐	☐	☐	☐	☐	☐
zervikale Dislokation (bis 100g)	☐	☐	☐	☐	☐	☐	☐
Intubation	☐	☐	☐	☐	☐	☐	☐
Vaginalabstrich	☐	☐	☐	☐	☐	☐	☐
mikrochirurgische Techniken	☐	☐	☐	☐	☐	☐	☐
Nahttechniken	☐	☐	☐	☐	☐	☐	☐

K4. **(Bedingung K3: Auswahl "sonstige Methoden") Bitte beschreiben Sie sonstige Methoden kurz.**

K5.b **Wie gut sind folgende anatomische Merkmale an Rat simulator C ausgebildet?**

Bitte vergeben Sie Schulnoten von 1 bis 6:

1: sehr gut; 2: gut; 3: befriedigend; 4: ausreichend; 5: mangelhaft; 6: ungenügend.

	1	2	3	4	5	6	keine Angabe
Haptik allgemein	☐	☐	☐	☐	☐	☐	☐
⇒ speziell: Verschieblichkeit und Konsistenz der Haut	☐	☐	☐	☐	☐	☐	☐
Körpergröße	☐	☐	☐	☐	☐	☐	☐
Gewicht	☐	☐	☐	☐	☐	☐	☐
Körperform	☐	☐	☐	☐	☐	☐	☐
Proportionen	☐	☐	☐	☐	☐	☐	☐
Vorhandensein geschlechtsspezifischer Merkmale	☐	☐	☐	☐	☐	☐	☐
Vorhandensein von Blutgefäßen	☐	☐	☐	☐	☐	☐	☐
Lage der Blutgefäße	☐	☐	☐	☐	☐	☐	☐
Verlauf der Blutgefäße	☐	☐	☐	☐	☐	☐	☐
Beschaffenheit der Blutgefäße	☐	☐	☐	☐	☐	☐	☐
Vorhandensein von Gelenken	☐	☐	☐	☐	☐	☐	☐
Beweglichkeit der Gelenke	☐	☐	☐	☐	☐	☐	☐

K6. **Wie praktikabel ist Rat simulator C im Einsatz mehrerer Kurse?**

Bitte geben Sie an, wie gut folgende Aussagen über Rat simulator C zutreffen.

	trifft voll und ganz zu	trifft weitgehend zu	trifft eher zu	trifft eher nicht zu	trifft weitgehend nicht zu	trifft überhaupt nicht zu	keine Angabe
Die Gebrauchsanweisung ist ausführlich und verständlich.	☐	☐	☐	☐	☐	☐	☐
Den Simulator kann man gut lagern und transportieren.	☐	☐	☐	☐	☐	☐	☐
Den Simulator kann man gut reinigen und desinfizieren.	☐	☐	☐	☐	☐	☐	☐
Der Simulator ist aus desinfektionsbeständigem Material.	☐	☐	☐	☐	☐	☐	☐
Der Simulator ist aus robustem Material.	☐	☐	☐	☐	☐	☐	☐
Der Simulator ist aus realitätsnahem Material.	☐	☐	☐	☐	☐	☐	☐
Die Anschaffungskosten sind angemessen.	☐	☐	☐	☐	☐	☐	☐
Die Lebensdauer ist angemessen.	☐	☐	☐	☐	☐	☐	☐
Laufende Kosten für Ersatzteile und Verbrauchsmaterialien sind angemessen.	☐	☐	☐	☐	☐	☐	☐
Die Ersatzteile haben eine lange Lebensdauer.	☐	☐	☐	☐	☐	☐	☐
Ersatzteile gibt es für alle Methoden, die am Simulator geübt werden.	☐	☐	☐	☐	☐	☐	☐
Die Ersatzteile lassen sich leicht austauschen.	☐	☐	☐	☐	☐	☐	☐

Teil L: Rat simulator D (Bedingung E1 Auswahl "sporadische Nutzung" oder "regelmäßige Nutzung" für Rat simulator D)

L1.b **Seit wie vielen Jahren wird Rat simulator D in Ihrem Kurs eingesetzt?**

Bitte geben Sie nur Zahlen ein.

seit etwa ☐☐☐☐ Jahren

L2.b Wie zufrieden sind Sie mit Rat simulator D insgesamt?

- sehr zufrieden ☐
- ziemlich zufrieden ☐
- eher zufrieden ☐
- eher unzufrieden ☐
- ziemlich unzufrieden ☐
- sehr unzufrieden ☐
- keine Angabe ☐

L3. **Wie gut können folgende Methoden an Rat simulator D geübt werden?**

Bitte vergeben Sie Schulnoten von 1 bis 6:

1: sehr gut; 2: gut; 3: befriedigend; 4: ausreichend; 5: mangelhaft; 6: ungenügend; kein Training: Methode wird nicht am Simulator tainiert.

	1	2	3	4	5	6	kein Training
Handling	☐	☐	☐	☐	☐	☐	☐
Fixation	☐	☐	☐	☐	☐	☐	☐
Ohrlochmarkierung	☐	☐	☐	☐	☐	☐	☐
Applikation per os mittels Sonde	☐	☐	☐	☐	☐	☐	☐
Applikation per os ohne Sonde	☐	☐	☐	☐	☐	☐	☐
Injektion subkutan	☐	☐	☐	☐	☐	☐	☐
Injektion intramuskulär	☐	☐	☐	☐	☐	☐	☐
Injektion intraperitoneal	☐	☐	☐	☐	☐	☐	☐
Injektion intravenös dorsale Penisvene (Ratte)	☐	☐	☐	☐	☐	☐	☐
Injektion intravenös Vena caudalis mediana	☐	☐	☐	☐	☐	☐	☐
Blutentnahme Vena sublingualis (Ratte)	☐	☐	☐	☐	☐	☐	☐
Blutentnahme Vena facialis (Maus)	☐	☐	☐	☐	☐	☐	☐
Blutentnahme retrobulbärer Venenplexus	☐	☐	☐	☐	☐	☐	☐
Blutentnahme Vena saphena	☐	☐	☐	☐	☐	☐	☐
Blutentnahme Vena caudalis mediana	☐	☐	☐	☐	☐	☐	☐
Blutentnahme intrakardial	☐	☐	☐	☐	☐	☐	☐
zervikale Dislokation (bis 100g)	☐	☐	☐	☐	☐	☐	☐
Intubation	☐	☐	☐	☐	☐	☐	☐
Vaginalabstrich	☐	☐	☐	☐	☐	☐	☐
mikrochirurgische Techniken	☐	☐	☐	☐	☐	☐	☐
Nahttechniken	☐	☐	☐	☐	☐	☐	☐
sonstige Methoden	☐	☐	☐	☐	☐	☐	☐

L4. **(Bedingung L3: Auswahl "sonstige Methoden") Bitte beschreiben Sie sonstige Methoden kurz.**

L5. **Wie gut sind folgende anatomische Merkmale an Rat simulator D nachgebildet?**

Bitte vergeben Sie Schulnoten von 1 bis 6:

1: sehr gut; 2: gut; 3: befriedigend; 4: ausreichend; 5: mangelhaft; 6: ungenügend.

	1	2	3	4	5	6	keine Angabe
Haptik allgemein	☐	☐	☐	☐	☐	☐	☐
⇒ speziell: Verschieblichkeit und Konsistenz der Haut	☐	☐	☐	☐	☐	☐	☐
Körpergröße	☐	☐	☐	☐	☐	☐	☐
Gewicht	☐	☐	☐	☐	☐	☐	☐
Körperform	☐	☐	☐	☐	☐	☐	☐
Proportionen	☐	☐	☐	☐	☐	☐	☐
Vorhandensein geschlechtsspezifischer Merkmale	☐	☐	☐	☐	☐	☐	☐
Vorhandensein von Blutgefäßen	☐	☐	☐	☐	☐	☐	☐
Lage der Blutgefäße	☐	☐	☐	☐	☐	☐	☐
Verlauf der Blutgefäße	☐	☐	☐	☐	☐	☐	☐
Beschaffenheit der Blutgefäße	☐	☐	☐	☐	☐	☐	☐
Vorhandensein von Gelenken	☐	☐	☐	☐	☐	☐	☐
Beweglichkeit der Gelenke	☐	☐	☐	☐	☐	☐	☐

L6. Wie praktikabel ist Rat simulator D im Einsatz mehrerer Kurse?

Bitte geben Sie an, wie gut folgende Aussagen über Rat simulator D zutreffen.

	trifft voll und ganz zu	trifft weitgehend zu	trifft eher zu	trifft eher nicht zu	trifft weitgehend nicht zu	trifft überhaupt nicht zu	keine Angabe
Die Gebrauchsanweisung ist ausführlich und verständlich.	☐	☐	☐	☐	☐	☐	☐
Den Simulator kann man gut lagern und transportieren.	☐	☐	☐	☐	☐	☐	☐
Den Simulator kann man gut reinigen und desinfizieren.	☐	☐	☐	☐	☐	☐	☐
Der Simulator ist aus desinfektionsbeständigem Material.	☐	☐	☐	☐	☐	☐	☐
Der Simulator ist aus robustem Material.	☐	☐	☐	☐	☐	☐	☐
Der Simulator ist aus realitätsnahem Material.	☐	☐	☐	☐	☐	☐	☐
Die Anschaffungskosten sind angemessen.	☐	☐	☐	☐	☐	☐	☐
Die Lebensdauer ist angemessen.	☐	☐	☐	☐	☐	☐	☐
Laufende Kosten für Ersatzteile und Verbrauchsmaterialien sind angemessen.	☐	☐	☐	☐	☐	☐	☐
Die Ersatzteile haben eine lange Lebensdauer.	☐	☐	☐	☐	☐	☐	☐
Ersatzteile gibt es für alle Methoden, die am Simulator geübt werden.	☐	☐	☐	☐	☐	☐	☐
Die Ersatzteile lassen sich leicht austauschen.	☐	☐	☐	☐	☐	☐	☐

Teil M: Rat simulator E Bedingung E1 Auswahl "sporadische Nutzung " oder "regelmäßige Nutzung" für Rat simulator E)

M1. Seit wie vielen Jahren wird Rat simulator E in Ihrem Kurs eingesetzt?

Bitte geben Sie nur Zahlen ein.

seit etwa ☐☐☐☐ Jahren

M2. Wie zufrieden sind Sie mit Rat simulator E insgesamt?

- sehr zufrieden ☐
- ziemlich zufrieden ☐
- eher zufrieden ☐
- eher unzufrieden ☐
- ziemlich unzufrieden ☐
- sehr unzufrieden ☐
- keine Angabe ☐

M3. Wie gut können folgende Methoden an Rat simulator E geübt werden?

Bitte vergeben Sie Schulnoten von 1 bis 6:

1: sehr gut; 2: gut; 3: befriedigend; 4: ausreichend; 5: mangelhaft; 6: ungenügend; kein Training: Methode wird nicht am Simulator trainiert.

	1	2	3	4	5	6	kein Training
Handling	☐	☐	☐	☐	☐	☐	☐
Fixation	☐	☐	☐	☐	☐	☐	☐
Ohrlochmarkierung	☐	☐	☐	☐	☐	☐	☐
Applikation per os mittels Sonde	☐	☐	☐	☐	☐	☐	☐
Applikation per os ohne Sonde	☐	☐	☐	☐	☐	☐	☐
Injektion subkutan	☐	☐	☐	☐	☐	☐	☐
Injektion intramuskulär	☐	☐	☐	☐	☐	☐	☐
Injektion intraperitoneal	☐	☐	☐	☐	☐	☐	☐
Injektion intravenös dorsale Penisvene (Ratte)	☐	☐	☐	☐	☐	☐	☐
Injektion intravenös Vena caudalis mediana	☐	☐	☐	☐	☐	☐	☐
Blutentnahme Vena sublingualis (Ratte)	☐	☐	☐	☐	☐	☐	☐
Blutentnahme Vena facialis (Maus)	☐	☐	☐	☐	☐	☐	☐
Blutentnahme retrobulbärer Venenplexus	☐	☐	☐	☐	☐	☐	☐
Blutentnahme Vena saphena	☐	☐	☐	☐	☐	☐	☐
Blutentnahme Vena caudalis mediana	☐	☐	☐	☐	☐	☐	☐
Blutentnahme intrakardial	☐	☐	☐	☐	☐	☐	☐
zervikale Dislokation (bis 100g)	☐	☐	☐	☐	☐	☐	☐
Intubation	☐	☐	☐	☐	☐	☐	☐
Vaginalabstrich	☐	☐	☐	☐	☐	☐	☐
mikrochirurgische Techniken	☐	☐	☐	☐	☐	☐	☐
Nahttechniken	☐	☐	☐	☐	☐	☐	☐
sonstige Methoden	☐	☐	☐	☐	☐	☐	☐

M4. **(Bedingung M3: Auswahl "sonstige Methoden") Bitte beschreiben Sie sonstige Methoden kurz.**

M5. **Wie gut sind folgende anatomische Merkmale an Rat simulator E nachgebildet?**

Bitte vergeben Sie Schulnoten von 1 bis 6:

1: sehr gut; 2: gut; 3: befriedigend; 4: ausreichend; 5: mangelhaft; 6: ungenügend.

	1	2	3	4	5	6	keine Angabe
Haptik allgemein	☐	☐	☐	☐	☐	☐	☐
⇒ speziell: Verschieblichkeit und Konsistenz der Haut	☐	☐	☐	☐	☐	☐	☐
Körpergröße	☐	☐	☐	☐	☐	☐	☐
Gewicht	☐	☐	☐	☐	☐	☐	☐
Körperform	☐	☐	☐	☐	☐	☐	☐
Proportionen	☐	☐	☐	☐	☐	☐	☐
Vorhandensein geschlechtsspezifischer Merkmale	☐	☐	☐	☐	☐	☐	☐
Vorhandensein von Blutgefäßen	☐	☐	☐	☐	☐	☐	☐
Lage der Blutgefäße	☐	☐	☐	☐	☐	☐	☐
Verlauf der Blutgefäße	☐	☐	☐	☐	☐	☐	☐
Beschaffenheit der Blutgefäße	☐	☐	☐	☐	☐	☐	☐
Vorhandensein von Gelenken	☐	☐	☐	☐	☐	☐	☐
Beweglichkeit der Gelenke	☐	☐	☐	☐	☐	☐	☐

M6. Wie praktikabel ist Rat simulator E im Einsatz mehrerer Kurse?

Bitte geben Sie an wie gut folgende Aussagen über Rat simulator E zutreffen.

	trifft voll und ganz zu	trifft weitgehend zu	trifft eher zu	trifft eher nicht zu	trifft weitgehend nicht zu	trifft überhaupt nicht zu	keine Angabe
Die Gebrauchsanweisung ist ausführlich und verständlich.	☐	☐	☐	☐	☐	☐	☐
Den Simulator kann man gut lagern und transportieren.	☐	☐	☐	☐	☐	☐	☐
Den Simulator kann man gut reinigen und desinfizieren.	☐	☐	☐	☐	☐	☐	☐
Der Simulator ist aus desinfektionsbeständigem Material.	☐	☐	☐	☐	☐	☐	☐
Der Simulator ist aus robustem Material.	☐	☐	☐	☐	☐	☐	☐
Der Simulator ist aus realitätsnahem Material.	☐	☐	☐	☐	☐	☐	☐
Die Anschaffungskosten sind angemessen.	☐	☐	☐	☐	☐	☐	☐
Die Lebensdauer ist angemessen.	☐	☐	☐	☐	☐	☐	☐
Laufende Kosten für Ersatzteile und Verbrauchsmaterialien sind angemessen.	☐	☐	☐	☐	☐	☐	☐
Die Ersatzteile haben eine lange Lebensdauer.	☐	☐	☐	☐	☐	☐	☐
Ersatzteile gibt es für alle Methoden, die am Simulator geübt werden.	☐	☐	☐	☐	☐	☐	☐
Die Ersatzteile lassen sich leicht austauschen.	☐	☐	☐	☐	☐	☐	☐

Teil N: Mouse simulator (Bedingung E1 Auswahl "sporadische Nutzung " oder "regelmäßige Nutzung" für Mouse simulator)

N1. Seit wie vielen Jahren wird Mouse simulator in Ihrem Kurs eingesetzt?

Bitte geben Sie nur Zahlen ein.

seit etwa ☐☐☐☐ Jahren

N2. Wie zufrieden sind Sie mit Mouse simulator insgesamt?

- sehr zufrieden ☐
- ziemlich zufrieden ☐
- eher zufrieden ☐
- eher unzufrieden ☐
- ziemlich unzufrieden ☐
- sehr unzufrieden ☐
- keine Angabe ☐

N3. Wie gut können folgende Methoden an Mouse simulator geübt werden?

Bitte vergeben Sie Schulnoten von 1 bis 6:

1: sehr gut; 2: gut; 3: befriedigend; 4: ausreichend; 5: mangelhaft; 6: ungenügend; kein Training: Methode wird nicht am Simulator trainiert.

	1	2	3	4	5	6	kein Training
Handling	☐	☐	☐	☐	☐	☐	☐
Fixation	☐	☐	☐	☐	☐	☐	☐
Ohrlochmarkierung	☐	☐	☐	☐	☐	☐	☐
Applikation per os mittels Sonde	☐	☐	☐	☐	☐	☐	☐
Applikation per os ohne Sonde	☐	☐	☐	☐	☐	☐	☐
Injektion subkutan	☐	☐	☐	☐	☐	☐	☐
Injektion intramuskulär	☐	☐	☐	☐	☐	☐	☐
Injektion intraperitoneal	☐	☐	☐	☐	☐	☐	☐
Injektion intravenös dorsale Penisvene (Ratte)	☐	☐	☐	☐	☐	☐	☐
Injektion intravenös Vena caudalis mediana	☐	☐	☐	☐	☐	☐	☐
Blutentnahme Vena sublingualis (Ratte)	☐	☐	☐	☐	☐	☐	☐
Blutentnahme Vena facialis (Maus)	☐	☐	☐	☐	☐	☐	☐
Blutentnahme retrobulbärer Venenplexus	☐	☐	☐	☐	☐	☐	☐
Blutentnahme Vena saphena	☐	☐	☐	☐	☐	☐	☐
Blutentnahme Vena caudalis mediana	☐	☐	☐	☐	☐	☐	☐
Blutentnahme intrakardial	☐	☐	☐	☐	☐	☐	☐
zervikale Dislokation (bis 100g)	☐	☐	☐	☐	☐	☐	☐
Intubation	☐	☐	☐	☐	☐	☐	☐
Vaginalabstrich	☐	☐	☐	☐	☐	☐	☐
mikrochirurgische Techniken	☐	☐	☐	☐	☐	☐	☐
Nahttechniken	☐	☐	☐	☐	☐	☐	☐
sonstige Methoden	☐	☐	☐	☐	☐	☐	☐

N4. (Bedingung N3: Auswahl "sonstige Methoden") Bitte beschreiben Sie sonstige Methoden kurz.

N5. Wie gut sind folgende anatomische Merkmale an Mouse simulator nachgebildet?

Bitte vergeben Sie Schulnoten von 1 bis 6:

1: sehr gut; 2: gut; 3: befriedigend; 4: ausreichend; 5: mangelhaft; 6: ungenügend.

	1	2	3	4	5	6	keine Angabe
Haptik allgemein	☐	☐	☐	☐	☐	☐	☐
⇒ speziell: Verschieblichkeit und Konsistenz der Haut	☐	☐	☐	☐	☐	☐	☐
Körpergröße	☐	☐	☐	☐	☐	☐	☐
Gewicht	☐	☐	☐	☐	☐	☐	☐
Körperform	☐	☐	☐	☐	☐	☐	☐
Proportionen	☐	☐	☐	☐	☐	☐	☐
Vorhandensein geschlechtsspezifischer Merkmale	☐	☐	☐	☐	☐	☐	☐
Vorhandensein von Blutgefäßen	☐	☐	☐	☐	☐	☐	☐
Lage der Blutgefäße	☐	☐	☐	☐	☐	☐	☐
Verlauf der Blutgefäße	☐	☐	☐	☐	☐	☐	☐
Beschaffenheit der Blutgefäße	☐	☐	☐	☐	☐	☐	☐
Vorhandensein von Gelenken	☐	☐	☐	☐	☐	☐	☐
Beweglichkeit der Gelenke	☐	☐	☐	☐	☐	☐	☐

N6. Wie praktikabel ist Mouse simulator im Einsatz mehrerer Kurse?

Bitte geben Sie an, wie gut folgende Aussagen über Mouse simulator zutreffen.

	trifft voll und ganz zu	trifft weitgehend zu	trifft eher zu	trifft eher nicht zu	Trifft weitgehend nicht zu	trifft überhaupt nicht zu	keine Angabe
Die Gebrauchsanweisung ist ausführlich und verständlich.	☐	☐	☐	☐	☐	☐	☐
Den Simulator kann man gut lagern und transportieren.	☐	☐	☐	☐	☐	☐	☐
Den Simulator kann man gut reinigen und desinfizieren.	☐	☐	☐	☐	☐	☐	☐
Der Simulator ist aus desinfektionsbeständigem Material.	☐	☐	☐	☐	☐	☐	☐
Der Simulator ist aus robustem Material.	☐	☐	☐	☐	☐	☐	☐
Der Simulator ist aus realitätsnahem Material.	☐	☐	☐	☐	☐	☐	☐
Die Anschaffungskosten sind angemessen.	☐	☐	☐	☐	☐	☐	☐
Die Lebensdauer ist angemessen.	☐	☐	☐	☐	☐	☐	☐
Laufende Kosten für Ersatzteile und Verbrauchsmaterialien sind angemessen.	☐	☐	☐	☐	☐	☐	☐
Die Ersatzteile haben eine lange Lebensdauer.	☐	☐	☐	☐	☐	☐	☐
Ersatzteile gibt es für alle Methoden, die am Simulator geübt werden.	☐	☐	☐	☐	☐	☐	☐
Die Ersatzteile lassen sich leicht austauschen.	☐	☐	☐	☐	☐	☐	☐

Teil O: Rat simulator F (Bedingung E1 Auswahl "sporadische Nutzung " oder "regelmäßige Nutzung" für Rat simulator F)

O1. Seit wie vielen Jahren wird Rat simulator F in Ihrem Kurs eingesetzt?

Bitte geben Sie nur Zahlen ein.

seit etwa ☐☐☐☐ Jahren

O2. Wie zufrieden sind Sie mit Rat simulator F ingesamt?

- sehr zufrieden ☐
- ziemlich zufrieden ☐
- eher zufrieden ☐
- eher unzufrieden ☐
- ziemlich unzufrieden ☐
- sehr unzufrieden ☐
- keine Angabe ☐

O3. Wie gut können folgende Methoden an Rat simulator F geübt werden?

Bitte vergeben Sie Schulnoten von 1 bis 6:

1: sehr gut; 2: gut; 3: befriedigend; 4: ausreichend; 5: mangelhaft; 6: ungenügend; kein Training: Methode wird nicht am Simulator trainiert.

	1	2	3	4	5	6	kein Training
mikrochirurgische Techniken	☐	☐	☐	☐	☐	☐	☐
Nahttechniken	☐	☐	☐	☐	☐	☐	☐
sonstige Methoden	☐	☐	☐	☐	☐	☐	☐

O4. (Bedingung O3: Auswahl "sonstige Methoden") Bitte beschreiben Sie sonstige Methoden kurz.

O5. Wie gut sind folgende anatomische Merkmale an Rat simulator F nachgebildet?

Bitte vergeben Sie Schulnoten von 1 bis 6:

1: sehr gut; 2: gut; 3: befriedigend; 4: ausreichend; 5: mangelhaft; 6: ungenügend.

	1	2	3	4	5	6	keine Angabe
Haptik allgemein	☐	☐	☐	☐	☐	☐	☐
⇒ speziell: Konsistenz der Haut	☐	☐	☐	☐	☐	☐	☐
Körpergröße	☐	☐	☐	☐	☐	☐	☐
Körperform	☐	☐	☐	☐	☐	☐	☐
Proportionen	☐	☐	☐	☐	☐	☐	☐
Vorhandensein geschlechtsspezifischer Merkmale	☐	☐	☐	☐	☐	☐	☐
Vorhandensein von Blutgefäßen	☐	☐	☐	☐	☐	☐	☐
Lage der Blutgefäße	☐	☐	☐	☐	☐	☐	☐
Verlauf der Blutgefäße	☐	☐	☐	☐	☐	☐	☐
Beschaffenheit der Blutgefäße	☐	☐	☐	☐	☐	☐	☐
Lage der Niere	☐	☐	☐	☐	☐	☐	☐
Beschaffenheit der Niere	☐	☐	☐	☐	☐	☐	☐
Form der Niere	☐	☐	☐	☐	☐	☐	☐
Lage der Blase	☐	☐	☐	☐	☐	☐	☐
Beschaffenheit der Blase	☐	☐	☐	☐	☐	☐	☐
Form der Blase	☐	☐	☐	☐	☐	☐	☐
Vorhandensein der Luftröhre	☐	☐	☐	☐	☐	☐	☐
Lage der Luftröhre	☐	☐	☐	☐	☐	☐	☐
Beschaffenheit der Luftröhre	☐	☐	☐	☐	☐	☐	☐
Vorhandensein der Speiseröhre	☐	☐	☐	☐	☐	☐	☐
Lage der Speiseröhre	☐	☐	☐	☐	☐	☐	☐
Beschaffenheit der Speiseröhre	☐	☐	☐	☐	☐	☐	☐

O6. Wie praktikabel ist Rat simulator F im Einsatz mehrerer Kurse?

Bitte geben Sie an, wie gut folgende Aussagen über Rat simulator F zutreffen.

	trifft voll und ganz zu	trifft weitgehend zu	trifft eher zu	trifft eher nicht zu	trifft weitgehend nicht zu	trifft überhaupt nicht zu	keine Angabe
Die Gebrauchsanweisung ist ausführlich und verständlich.	☐	☐	☐	☐	☐	☐	☐
Den Simulator kann man gut lagern und transportieren.	☐	☐	☐	☐	☐	☐	☐
Den Simulator kann man gut reinigen und desinfizieren.	☐	☐	☐	☐	☐	☐	☐
Der Simulator ist aus desinfektionsbeständigem Material.	☐	☐	☐	☐	☐	☐	☐
Der Simulator ist aus robustem Material.	☐	☐	☐	☐	☐	☐	☐
Der Simulator ist aus realitätsnahem Material.	☐	☐	☐	☐	☐	☐	☐
Die Anschaffungskosten sind angemessen.	☐	☐	☐	☐	☐	☐	☐
Die Lebensdauer ist angemessen.	☐	☐	☐	☐	☐	☐	☐
Laufende Kosten für Ersatzteile und Verbrauchsmaterialien sind angemessen.	☐	☐	☐	☐	☐	☐	☐
Die Ersatzteile haben eine lange Lebensdauer.	☐	☐	☐	☐	☐	☐	☐
Ersatzteile gibt es für alle Methoden, die am Simulator geübt werden.	☐	☐	☐	☐	☐	☐	☐
Die Ersatzteile lassen sich leicht austauschen.	☐	☐	☐	☐	☐	☐	☐

Teil P: Methodische Anforderungen an einen neuen Simulator

Im letzten Teil der Umfrage geht es um Ihre Ansprüche an einen neuen Simulator. Die Fragen gliedern sich in methodische, anatomische und praktische Anforderungen.

1. Methodische Anforderungen an einen neuen Simulator

⇒ Welche Methoden sind Ihnen wichtig?

P1. Welche Methoden möchten Sie an einem neuen Rattensimulator trainieren?

Ordnen Sie maximal 10 Methoden in die rechte Liste ein (höchste Priorität oben). Ein Doppelklick verschiebt ein Element in die andere Liste. Die Elemente können mit der Maus verschoben werden.

Handling	☐☐
Fixation	☐☐
Ohrlochmarkierung	☐☐
Applikation per os mittels Sonde	☐☐
Applikation per os ohne Sonde	☐☐
Injektion subkutan	☐☐
Injektion intramuskulär	☐☐
Injektion intraperitoneal	☐☐
Injektion intravenös dorsale Penisvene	☐☐
Injektion intravenös Vena caudalis mediana	☐☐
Blutentnahme Vena sublingualis	☐☐
Blutentnahme retrobulbärer Venenplexus	☐☐
Blutentnahme Vena saphena	☐☐
Blutentnahme Vena caudalis mediana	☐☐
Blutentnahme intrakardial	☐☐
zervikale Dislokation (bis 100g)	☐☐
Intubation	☐☐
Vaginalabstrich	☐☐
Nahttechniken	☐☐
sonstige Methoden	☐☐

P2. (Bedingung P1 Auswahl "Sonstiges") Welche sonstigen Methoden möchten Sie am Rattensimulator trainieren?

P3. Welche Methoden möchten Sie an einem neuen Maussimulator trainieren?

Ordnen Sie maximal 10 Methoden in die rechte Liste ein (höchste Priorität oben).
Ein Doppelklick verschiebt ein Element in die andere Liste. Die Elemente können mit der Maus verschoben werden.

Handling

Fixation

Ohrlochmarkierung

Applikation per os mittels Sonde

Applikation per os ohne Sonde

Injektion subkutan

Injektion intramuskulär

Injektion intraperitoneal

Injektion intravenös Vena caudalis mediana

Blutentnahme Vena facialis

Blutentnahme retrobulbärer Venenplexus

Blutentnahme Vena saphena

Blutentnahme Vena caudalis mediana

Blutentnahme intrakardial

zervikale Dislokation (bis 100g)

Intubation

Vaginalabstrich

mikrochirurgische Techniken

Nahttechniken

sonstige Methoden

P4. (Bedingung P3: Auswahl "sonstige Methoden")
Welche sonstigen Methoden möchten Sie am Maussimulator trainieren?

Teil Q: Anatomische Anforderungen an einen neuen Simulator

2. Anatomische Anforderungen an einen neuen Simulator

⇒ Welche Strukturen müssen Ihrer Meinung nach möglichst lebensgetreu nachgebildet werden?

Q1. Wie wichtig ist Ihnen die möglichst korrekte Nachbildung anatomischer Verhältnisse insgesamt?

- sehr wichtig ☐
- ziemlich wichtig ☐
- eher wichtig ☐
- eher unwichtig ☐
- ziemlich unwichtig ☐
- sehr unwichtig ☐
- keine Angabe ☐

Q2. Wie wichtig ist Ihnen die möglichst korrekte Nachbildung allgemeiner anatomischer Merkmale?

	sehr wichtig	ziemlich wichtig	eher wichtig	eher unwichtig	ziemlich unwichtig	sehr unwichtig	keine Angabe
Haptik allgemein	☐	☐	☐	☐	☐	☐	☐
⇒ speziell: Verschieblichkeit und Konsistenz der Haut	☐	☐	☐	☐	☐	☐	☐
Körpergröße	☐	☐	☐	☐	☐	☐	☐
Gewicht	☐	☐	☐	☐	☐	☐	☐
Körperform	☐	☐	☐	☐	☐	☐	☐
Proportionen	☐	☐	☐	☐	☐	☐	☐
Vorhandensein geschlechtsspezifischer Merkmale	☐	☐	☐	☐	☐	☐	☐
Vorhandensein von Blutgefäßen	☐	☐	☐	☐	☐	☐	☐
Lage der Blutgefäße	☐	☐	☐	☐	☐	☐	☐
Verlauf der Blutgefäße	☐	☐	☐	☐	☐	☐	☐
Beschaffenheit der Blutgefäße	☐	☐	☐	☐	☐	☐	☐
Vorhandensein von Gelenken	☐	☐	☐	☐	☐	☐	☐
Beweglichkeit der Gelenke	☐	☐	☐	☐	☐	☐	☐

Q3.Wie wichtig ist ihnen die möglichst korrekte Nachbildung der Kopf-Hals-Region?

	sehr wichtig	ziemlich wichtig	eher wichtig	eher unwichtig	ziemlich unwichtig	sehr unwichtig	keine Angabe
Sinnesorgane allgemein	☐	☐	☐	☐	☐	☐	☐
⇒ speziell Augen	☐	☐	☐	☐	☐	☐	☐
⇒ speziell Ohren	☐	☐	☐	☐	☐	☐	☐
⇒ speziell Nase	☐	☐	☐	☐	☐	☐	☐
⇒ speziell Vibrissen	☐	☐	☐	☐	☐	☐	☐
⇒ speziell Haut	☐	☐	☐	☐	☐	☐	☐
Organe der Maulhöhle allgemein	☐	☐	☐	☐	☐	☐	☐
⇒ speziell Zähne	☐	☐	☐	☐	☐	☐	☐
⇒ speziell Zunge	☐	☐	☐	☐	☐	☐	☐
⇒ speziell Kiefergelenk	☐	☐	☐	☐	☐	☐	☐
⇒ speziell Kehlkopf	☐	☐	☐	☐	☐	☐	☐
Vorhandensein der Luftröhre	☐	☐	☐	☐	☐	☐	☐
Lage der Luftröhre	☐	☐	☐	☐	☐	☐	☐
Beschaffenheit der Luftröhre	☐	☐	☐	☐	☐	☐	☐
Vorhandensein der Speiseröhre	☐	☐	☐	☐	☐	☐	☐
Lage der Speiseröhre	☐	☐	☐	☐	☐	☐	☐
Beschaffenheit der Speiseröhre	☐	☐	☐	☐	☐	☐	☐
anderes Merkmal / andere Merkmale	☐	☐	☐	☐	☐	☐	☐

Q4. (Bedingung Q3 Auswahl "anderes Merkmal/andere Merkmale") Welches andere Merkmal der Kopf-Hals-Region ist Ihnen wichtig?

Q5. (Bedingung Q3 Auswahl "anderes Merkmal/andere Merkmale") Welches andere Merkmal der Kopf-Hals-Region ist Ihnen unwichtig?

Teil R: Anatomische Anforderungen an einen neuen Simulator

2. Anatomische Anforderungen an einen neuen Simulator

⇒Welche Strukturen sind Ihnen wichtig?

R1. **Wie wichtig ist Ihnen das Vorhandensein folgender Nachbildungen der Sinnesorgane?**

Bitte ordnen Sie die Sinnesorgane nach ihrer Wichtigkeit (höchste Priorität oben). Falls ein Sinnesorgan unwichtig ist, belassen Sie es bitte auf der linken Seite. Ein Doppelklick verschiebt ein Element auf die andere Seite. Die Elemente können mit der Maus verschoben werden.

Augen ☐

Ohren ☐

Nase ☐

Vibrissen ☐

Haut ☐

R2. **Wie wichtig ist Ihnen das Vorhandensein folgender Nachbildungen der Organe der Maulhöhle und des Halses?**

Bitte ordnen Sie die Organe nach ihrer Wichtigkeit (höchste Prioritätb oben). Falls ein Organ unwichtig ist, belassen Sie es bitte auf der linken Seite. Ein Doppelklick verschiebt ein Element auf die andere Seite. Die Elemente können mit der Maus verschoben werden.

Zähne ☐

Zunge ☐

Kiefergelenk ☐

Kehlkopf ☐

Speiseröhre ☐

Luftröhre ☐

anderes Organ ☐

R3. (Bedingung R2: Auswahl "anderes Organ")
Welches andere Organ der Maulhöhle und des Halses ist Ihnen als Nachbildung wichtig?

R4. **Wie wichtig ist Ihnen das Vorhandensein folgender Blutgefäße bei einem Rattensimulator?**

Bitte ordnen Sie die Blutgefäße nach ihrer Wichtigkeit (höchste Priorität oben). Falls ein Blutgefäß unwichtig ist, belassen Sie es bitte auf der linken Seite. Ein Doppelklick verschiebt ein Element auf die andere Seite. Die Elemente können mit der Maus verschoben werden.

Vena sublingualis ☐

retrobulbärer Venenplexus ☐

Vena saphena ☐

Vena caudalis mediana ☐

anderes Gefäß ☐

R5. (Bedingung R4: Auswahl "anderes Gefäß")
Welches andere Blutgefäß ist Ihnen beim Rattensimulator wichtig?

R6.Wie wichtig ist Ihnen das Vorhandensein folgender Blutgefäße bei einem Maussimulator?

Bitte ordnen Sie die Blutgefäße nach ihrer Wichtigkeit (höchste Priorität oben). Falls ein Blutgefäß unwichtig ist, belassen Sie es bitte auf der linken Seite. Ein Doppelklick verschiebt ein Element auf die andere Seite. Die Elemente können mit der Maus verschoben werden.

Vena facialis ☐

retrobulbärer Venenplexus ☐

Vena saphena ☐

Vena caudalis mediana ☐

anderes Gefäß ☐

R7. (Bedingung R6: Auswahl "anderes Gefäß")
Welches andere Blutgefäß ist Ihnen beim Maussimulator wichtig?

Teil S: Praktische Anforderungen an einen neuen Simulator

3. Praktische Anforderungen an einen neuen Simulator

⇒ Welche Merkmale sind Ihnen beim Einsatz in mehreren Kursen wichtig?

S1. Wie wichtig sind Ihnen folgende Merkmale beim Einsatz in mehreren Kursen?

	sehr wichtig	ziemlich wichtig	eher wichtig	eher unwichtig	ziemlich unwichtig	sehr unwichtig	keine Angabe
Die Gebrauchsanweisung ist ausführlich und verständlich.	☐	☐	☐	☐	☐	☐	☐
Den Simulator kann man gut lagern und transportieren.	☐	☐	☐	☐	☐	☐	☐
Den Simulator kann man gut reinigen und desinfizieren.	☐	☐	☐	☐	☐	☐	☐
Der Simulator ist aus desinfektionsbeständigem Material.	☐	☐	☐	☐	☐	☐	☐
Der Simulator ist aus robusten Material.	☐	☐	☐	☐	☐	☐	☐
Der Simulator ist aus realitätsnahem Material.	☐	☐	☐	☐	☐	☐	☐
Die Anschaffungskosten sind angemessen.	☐	☐	☐	☐	☐	☐	☐
Die Lebensdauer ist angemessen.	☐	☐	☐	☐	☐	☐	☐
Laufende Kosten für Ersatzteile und Verbrauchsmaterialien sind angemessen.	☐	☐	☐	☐	☐	☐	☐
Die Ersatzteile haben eine lange Lebensdauer.	☐	☐	☐	☐	☐	☐	☐
Ersatzteile gibt es für alle Methoden, die am Simulator geübt werden.	☐	☐	☐	☐	☐	☐	☐
Die Ersatzteile lassen sich leicht austauschen.	☐	☐	☐	☐	☐	☐	☐

Teil T: Weitere Anforderungen an einen neuen Simulator

Sind Ihnen weitere Merkmale wichtig? Wir freuen uns auf Ihre Informationen!

T1. Welche weiteren Kriterien sind Ihnen bei einer Neuentwicklung wichtig?

methodische Anforderungen ☐☐☐☐☐☐☐☐☐☐

anatomische Anforderungen ☐☐☐☐☐☐☐☐☐☐

praktische Anforderungen ☐☐☐☐☐☐☐☐☐☐

Teil U: Fragen zu Ihrer Person

U1. Wie alt sind Sie?

☐☐

U2. Welches Geschlecht haben Sie?

- weiblich ☐
- männlich ☐
- divers ☐
- keine Angabe ☐

U3. Wie lange führen Sie schon versuchstierkundliche Kurse für Ratte und Maus durch?

☐☐☐☐☐

U4. Sind Sie Kursleiter/in oder Kursbetreuer/in?

- Ich bin Kursleiter/in. ☐
- Ich bin Kursbetreuer/in. ☐
- keine Angabe ☐
- Sonstiges ☐

Sonstiges

☐☐☐☐☐☐☐☐☐☐☐☐☐☐☐☐☐☐☐☐☐☐☐☐

U5. Anmerkungen, Lob & Kritik?

Hier können Sie mir Ihre Nachricht hinterlassen.

Wir bedanken uns herzlichst für Ihre Unterstützung!

Wir möchten Sie an dieser Stelle auf den zweiten Teil unserer Evaluierung aufmerksam machen: Die Evaluierung in Kursen - Eine Bewertung der Simulatoren aus Sicht der Kursteilnehmer/innen. Wenn Sie Interesse haben, mit Ihren Kursen an der Evaluierung aus Sicht der Kursteilnehmer/innen teilzunehmen oder weitere Informationen wünschen, können Sie über den Link https://survey.vetmed.fu-berlin.de/index.php/455897?lang=de einen Kontakt hinterlassen oder uns persönlich über *kontakt@simulRATor.de* anschreiben. Gerne senden wir Ihnen auf Wunsch auch eine Zusammenfassung der Studienergebnisse per E-Mail zu. Bitte schreiben Sie uns hierfür über *kontakt@simulRATor.de* an. *HINWEIS ZUM DATENSCHUTZ: Ihre Kontaktdaten können NICHT mit den Antworten der Fragen in Verbindung gebracht werden.* Sie können uns selbstverständlich jederzeit über *kontakt@simulRATor.de* Fragen stellen. Wir freuen uns auf Ihre Nachricht! Wir bedanken uns herzlichst für Ihre Unterstützung!

Melanie Humpenöder melanie.humpenoeder@fu-berlin.de **Institut für Tierschutz, Tierverhalten und Versuchstierkunde Königsweg 67 14163 Berlin** ***Giuliano Mario Corte giuliano.corte@fu-berlin.de*** **Institut für Veterinär-Anatomie Koserstraße 20 14059 Berlin Institut für Veterinär-Epidemiologie und Biometrie Königsweg 67 14163 Berlin**

Questionnaire for LAS course trainers and supervisors
Translated version of the PDF file of the original German questionnaire exported from free and open source online survey web app "Limesurvey" [28]. The questionaire was conducted online from 31 March 2018 until 30 June 2019 using "Limesurvey" [28]. Simulator product names were anonymized by coding (Rat simulator B, A, C, D, E, Mouse simulator and Rat simulator F) and illustrations were subsequently omitted in the PDF file. For illustrations of the simulators, see Table 1.

References

28. Schmitz, C. Homepage - LimeSurvey - simple online surveys. Available online: https://www.limesurvey.org/de/ (accessed on 30 March 2021).

Part A: Dear course trainers and supervisors,

in the Bf3R-funded cooperative project "SimulRATor" at the Department of Veterinary Medicine of Freie Universität Berlin, we, a team of staff members from the Institutes of Veterinary Anatomy, of Animal Welfare, Behavior and Laboratory Animal Science, and of Veterinary Epidemiology and Biometry, are developing an anatomical correct and cost-effective simulator for laboratory animal science courses.

In order to adapt the simulator for course implementation optimally, we evaluate the currently available rat and mouse simulators in the "SimulRATor" project from the perspective of the course trainers (including course supervisors) and the course participants according the current animal welfare regulations. In two anonymous online surveys, the use and satisfaction of simulators are recorded, as well as demands and needs for a new simulator.

The current survey is aimed at all carrying out laboratory animal science courses for rats and mice - with or without the use of simulators and is designed to assess simulators from the perspective of course trainers and course supervisors.

All results are incorporated into a final requirements analysis, on the basis of which a new simulator is created at the Institute of Veterinary Anatomy using 3D printing.

Please take about 15 minutes of your time. Your experience and commitment will help to develop a simulator that takes into account the demands of humans, animals and science alike.

Note on data protection: Your participation in the survey is voluntary. The survey data will be stored and administered anonymously. It is not possible to draw any conclusions about your participation. All data is managed in strict compliance with current data protection guidelines. Only project participants have access to the data and are bound to secrecy.
Under no circumstances will data be passed on to third parties. The data will only be used for research purposes. You can cancel the survey at any time without giving reasons and delete your data by closing the browser window.

Thank you very much for your participation!

Part B: General

Please use the "Back" and "Next" buttons to go back or forward.

The survey can be cached at any time under Continue Later. You can load your cached survey on the home page. Caching is recommended for interruptions longer than 30 minutes.

Please answer all of the following questions for the course you do most often.

B1. What courses for rat and/or mouse do you conduct?

- Course for persons performing animal experiments (former FELASA category B course) ☐
- Course for persons planning animal experiments (former FELASA category C course) ☐
- Other courses ☐
- none ☐

B2. How many courses do you conduct per year? Please enter only numbers.

Course for persons performing animal experiments (former FELASA category B course) [ ]

Course for persons planning animal experiments (former FELASA category C course) [ ]

Other courses [ ]

B3. What is the average number of participants and course instructors in your course?

Please enter numbers only.

Number of participants [ ]

course instructors (course trainers and course supervisors) [ ]

Part C: Alternative methods

C1. What methods do you use to supplement in the course?

- Instructions, drawings, photos ☐
- Videos ☐
- Learning software (on-/offline) ☐
- Computer simulations ☐
- Sof toys/dolls ☐
- Rat simulators ☐
- Mouse simulators ☐

Suture pads ☐

Others/do-it-yourself ☐

none ☐

C2. What other methods do you use? Condition selection of " others/do it yourself" C1.

Please describe them briefly.

☐

C3. How satisfied are you with your alternative methods?

	very satisfied	Quite satisfied	Rather satisfied	Rather dissatisfied	quite dissatisfied	Very dissatisfied	not specified
Instructions, drawings, photos	☐	☐	☐	☐	☐	☐	☐
Videos	☐	☐	☐	☐	☐	☐	☐
Learning software (on/offline)	☐	☐	☐	☐	☐	☐	☐
Computer simulations	☐	☐	☐	☐	☐	☐	☐
Soft toys / dolls	☐	☐	☐	☐	☐	☐	☐
Rat simulators	☐	☐	☐	☐	☐	☐	☐
Mouse simulators	☐	☐	☐	☐	☐	☐	☐
Suture pads	☐	☐	☐	☐	☐	☐	☐
Others/ do-it yourself	☐	☐	☐	☐	☐	☐	☐
none	☐	☐	☐	☐	☐	☐	☐

Part D (condition: C1 Selection of suture pads): Supplement with seam pads

D1. How many suture pads do you use in the course?

Seam pads ☐ Pcs.

D2. Since when have suture pads been used in your course?

since about ☐ Years

Part E: Simulators of the rat and mouse

E1. Which simulators are you familiar with? One answer for each possible.

	Not known	Theoretically known	Sporadic usage	Regular usage
Rat simulator B	☐	☐	☐	☐
Rat simulator A	☐	☐	☐	☐
Rat simulator C	☐	☐	☐	☐
Rat simulator D	☐	☐	☐	☐
Rat simulator E	☐	☐	☐	☐
Mouse simulator	☐	☐	☐	☐
Rat simulator F	☐	☐	☐	☐

E2. (Condition E1 selection "sporadic use" or “regular use” > 0).

How often do you work with the selected simulator(s)?

	never	in approx. 1/4 of all courses	in approx. 1/2 of all courses	in approx. 3/4 of all courses	in all courses	Other frequency
Rat simulator B	☐	☐	☐	☐	☐	☐
Rat simulator A	☐	☐	☐	☐	☐	☐
Rat simulator C	☐	☐	☐	☐	☐	☐
Rat simulator D	☐	☐	☐	☐	☐	☐
Rat simulator E	☐	☐	☐	☐	☐	☐
Mouse simulator	☐	☐	☐	☐	☐	☐
Rat simulator F	☐	☐	☐	☐	☐	☐

E3. (Condition E1 Select corresponding simulator)
In which frequency do you work with the selected simulator(s)?
Please indicate the answer in the ratio of all courses or use the field for a description.

Rat simulator B

Rat simulator A

Rat simulator C

Rat simulator D

Rat simulator E

Mouse simulator

Rat simulator F

E4. (Condition E1 Selection corresponding Simulator)
How many copies (= simulator per type) do you use in each course? Please enter numbers only.

Rat simulator B

Rat simulator A

Rat simulator C

Rat simulator D

Rat simulator E

Mouse simulator

Rat simulator F

E5. (E1 selection "sporadic use" or "regular use" corresponding simulator > 1)

Which of your simulators do you prefer to work with?

- Rat simulator B
- Rat simulator A
- Rat simulator C
- Rat simulator D
- Rat simulator E
- Mouse simulator
- Rat simulator F
- none
- all equal

Part F: (E1 selection "sporadic use" or “regular use" corresponding simulator > 0)
Supplementation with simulators

F1. How do you use the selected simulator(s) in the practical part of the course?

Multiple answers are possible.

- Demonstration of the techniques by supervisors incl. course trainers ☐
- Practice for participants on the simulator in a separate course unit ☐
- Practice for participants on the simulator parallel to the exercise on the animal ☐
- other ☐

F2. Must all course participants practice on the simulators or is the practice intended for individual course participants?

- Yes, the practice is mandatory. ☐
- Yes, the practice is voluntary. ☐
- No, the practice is intended for individual course participants. ☐
- Other ☐

Other

F3. Are you planning to purchase simulators for rat and/or mouse in the near future?

- Yes ☐
- No ☐

F4. Which simulator would you like to purchase?

Multiple answers are possible.

- The same simulator I already have : ☐
- Rat simulator B ☐
- Rat simulator A ☐
- Rat simulator C ☐
- Rat simulator D ☐
- Rat simulator E ☐
- Mouse simulator ☐

Rat simulator F ☐

a new/other simulator: ☐

new/other simulator: Rat Simulator B ☐

a new/other simulator: Rat Simulator A ☐

a new/different simulator: Rat Simulator C ☐

a new/other simulator: Rat Simulator D ☐

a new/other simulator: Rat Simulator E ☐

a new/other simulator: Mouse Simulator ☐

a new/other simulator: Rat Simulator F ☐

a new development ☐

a do-it-yourself ☐

other ☐

other

F5. Why don't you want to buy a simulator?

Multiple answers may be possible.

I do not plan to use simulators as a matter of principle. ☐

I don't have the resources for that at the moment. ☐

I have enough simulators. ☐

I am waiting for a new development. ☐

I invest in a do-it-yourself ☐

Other ☐

other

Part G: Transmissibility rat → mouse

G1. Can techniques for the mouse be trained on rat simulators?

Yes, all techniques on the mouse ☐

Yes, only certain techniques on the mouse ☐

No, no technique on the mouse ☐

G2. Which techniques for the mouse can be trained on rat simulators?

- Handling ☐
- Restraint ☐
- Ear hole marking ☐
- Application techniques ☐
- Injection subcutaneous ☐
- Injection intramuscular ☐
- Injection intraperitoneal ☐
- Injection intravenous vena caudalis mediana ☐
- Blood collection techniques ☐
- facial vein ☐
- retrobulbar venous plexus ☐
- saphenous vein ☐
- caudal vein mediana ☐
- intracardiac ☐
- other techniques ☐

G3. What other techniques for the mouse can be applied to Rat simulators to be trained?

Part H: The exercise on the animal

H1. Which methods on rat and/or mouse do you teach in the practical part of the course? Please indicate whether the exercise is performed on a conscious, anesthetized and/or dead rat or mouse.

Multiple answers are possible.

SQ001:

	Rat				Mouse			
	conscious	anesthetized	Post mortem	No training	conscious	anesthetized	Post mortem	No training
Handling	☐	☐	☐	☐	☐	☐	☐	☐
Restraint	☐	☐	☐	☐	☐	☐	☐	☐
Ear punching	☐	☐	☐	☐	☐	☐	☐	☐
Application per os with probe	☐	☐	☐	☐	☐	☐	☐	☐
Application per os without probe	☐	☐	☐	☐	☐	☐	☐	☐
Injection subcutaneous	☐	☐	☐	☐	☐	☐	☐	☐
Injection intramuscular	☐	☐	☐	☐	☐	☐	☐	☐
Injection intraperitoneal	☐	☐	☐	☐	☐	☐	☐	☐
Injection intravenously dorsal penile vein (rat)	☐	☐	☐	☐	☐	☐	☐	☐
Injection intravenous caudal vein mediana	☐	☐	☐	☐	☐	☐	☐	☐
Blood sampling sublingual vein (rat)	☐	☐	☐	☐	☐	☐	☐	☐
Blood collection facial vein (mouse)	☐	☐	☐	☐	☐	☐	☐	☐
Blood sampling retrobulbar venous plexus	☐	☐	☐	☐	☐	☐	☐	☐
Blood collection saphenous vein	☐	☐	☐	☐	☐	☐	☐	☐
Blood sampling caudal vein mediana	☐	☐	☐	☐	☐	☐	☐	☐
blood sampling intracardiac	☐	☐	☐	☐	☐	☐	☐	☐
cervical dislocation (up to 100g)	☐	☐	☐	☐	☐	☐	☐	☐
intubation	☐	☐	☐	☐	☐	☐	☐	☐
vaginal smear	☐	☐	☐	☐	☐	☐	☐	☐
microsurgical techniques	☐	☐	☐	☐	☐	☐	☐	☐
suture techniques	☐	☐	☐	☐	☐	☐	☐	☐
Other	☐	☐	☐	☐	☐	☐	☐	☐

H2. (condition H1 : Selection "Other")
What other methods are practiced in the practical part of the course?

Please describe them briefly.

Rat conscious	
Rat anesthetized	
Rat post mortem	
Mouse conscious	
Mouse anesthetized	
Mouse post mortem	

H3. How many live animals do the students practice on?
in the course? Please indicate the ratio of animals to course participants.

Number of animals:

Exercise on the rat	
Exercise on the mouse	

/ Course participants:

Exercise on the rat	
Exercise on the mouse	

Part I: Rat simulator B (condition E1 selection "sporadic use" or "regular use" for Rat simulator B)

I1. For how many years has Rat simulator B been used in your course?

Please enter numbers only.

for about ☐ years

I2. How satisfied are you with Rat simulator B overall?

- very satisfied ☐
- quite satisfied ☐
- rather satisfied ☐
- rather dissatisfied ☐
- quite dissatisfied ☐
- very dissatisfied ☐
- not specified ☐

I3. How well can the following methods be practiced on Rat simulator B?

Please assign school grades from 1 to 6:

1: very good; 2: good; 3: satisfactory; 4: sufficient; 5: poor; 6:insufficient; no training: method is not trained on the simulator.

	1	2	3	4	5	6	No
Handling	☐	☐	☐	☐	☐	☐	☐
Restraint	☐	☐	☐	☐	☐	☐	☐
Ear punching	☐	☐	☐	☐	☐	☐	☐
Application per os with probe	☐	☐	☐	☐	☐	☐	☐
Application per os without probe	☐	☐	☐	☐	☐	☐	☐
Injection subcutaneous	☐	☐	☐	☐	☐	☐	☐
Injection intramuscular	☐	☐	☐	☐	☐	☐	☐
Injection intraperitoneal	☐	☐	☐	☐	☐	☐	☐
Injection intravenously dorsal penile vein (rat)	☐	☐	☐	☐	☐	☐	☐
Injection intravenous caudal vein mediana	☐	☐	☐	☐	☐	☐	☐
Blood sampling sublingual vein (rat)	☐	☐	☐	☐	☐	☐	☐
Blood collection facial vein (mouse)	☐	☐	☐	☐	☐	☐	☐
Blood sampling retrobulbar venous plexus	☐	☐	☐	☐	☐	☐	☐
Blood collection saphenous vein	☐	☐	☐	☐	☐	☐	☐
Blood sampling caudal vein mediana	☐	☐	☐	☐	☐	☐	☐
blood sampling intracardiac	☐	☐	☐	☐	☐	☐	☐
cervical dislocation (up to 100g)	☐	☐	☐	☐	☐	☐	☐
intubation	☐	☐	☐	☐	☐	☐	☐
vaginal smear	☐	☐	☐	☐	☐	☐	☐
microsurgical techniques	☐	☐	☐	☐	☐	☐	☐
suture techniques	☐	☐	☐	☐	☐	☐	☐
Other	☐	☐	☐	☐	☐	☐	☐

I4. **(Condition I3: Select "other methods") Please briefly describe other methods.**

I5. **How well are the following anatomical features reproduced on Rat simulator B?**

Please assign school grades from 1 to 6:

1: very good; 2: good; 3: satisfactory; 4: sufficient; 5: poor; 6: insufficient.

	1	2	3	4	5	6	No
Haptics in general	☐	☐	☐	☐	☐	☐	☐
⇒ specially: displaceability and consistency of the skin	☐	☐	☐	☐	☐	☐	☐
Body size	☐	☐	☐	☐	☐	☐	☐
Weight	☐	☐	☐	☐	☐	☐	☐
Body shape	☐	☐	☐	☐	☐	☐	☐
Proportions	☐	☐	☐	☐	☐	☐	☐
Presence of gender-specific Features	☐	☐	☐	☐	☐	☐	☐
Presence of blood vessels	☐	☐	☐	☐	☐	☐	☐
Location of the blood vessels	☐	☐	☐	☐	☐	☐	☐
Course of the blood vessels	☐	☐	☐	☐	☐	☐	☐
Condition of the blood vessels	☐	☐	☐	☐	☐	☐	☐
Presence of joints	☐	☐	☐	☐	☐	☐	☐
Mobility of the joints	☐	☐	☐	☐	☐	☐	☐

I6. How practical is Rat simulator B in the use of multiple courses?

Please indicate how well the following statements are true about Rat simulator B.

	Fully applies	Largely applies	Rather applies	Rather does not apply	Largely does not apply	Does not apply at all	No answer
The manual is detailed and easy to understand.	☐	☐	☐	☐	☐	☐	☐
The simulator is easy to store and transport.	☐	☐	☐	☐	☐	☐	☐
The simulator is easy to clean and disinfect.	☐	☐	☐	☐	☐	☐	☐
The simulator is made of disinfection-resistant material.	☐	☐	☐	☐	☐	☐	☐
The simulator is made of robust material.	☐	☐	☐	☐	☐	☐	☐
The simulator is made of realistic material.	☐	☐	☐	☐	☐	☐	☐
The acquisition costs are reasonable.	☐	☐	☐	☐	☐	☐	☐
The service life is reasonable.	☐	☐	☐	☐	☐	☐	☐
Running costs for spare parts and consumables are reasonable.	☐	☐	☐	☐	☐	☐	☐
Spare parts have a long service life.	☐	☐	☐	☐	☐	☐	☐
Spare parts are available for all trainable methods on the simulator.	☐	☐	☐	☐	☐	☐	☐
Spare parts are easy to replace.	☐	☐	☐	☐	☐	☐	☐

Part J: Rat simulator A (condition E1 selection "sporadic use" or "regular use" for Rat simulator A)

J1. For how many years has the Rat simulator A been used in your course used?

Please enter numbers only.

for about ☐☐☐☐ years

J2. How satisfied are you with Rat simulator A overall?

- very satisfied ☐
- Quite satisfied ☐
- Rather satisfied ☐
- rather dissatisfied ☐
- quite dissatisfied ☐
- Very dissatisfied ☐
- not specified ☐

J3. How well can the following methods be practiced on Rat simulator A?

Please assign school grades from 1 to 6:

1: very good; 2: good; 3: satisfactory; 4: sufficient; 5: poor; 6: insufficient; no training: method is not trained on the simulator.

	1	2	3	4	5	6	No
Handling	☐	☐	☐	☐	☐	☐	☐
Restraint	☐	☐	☐	☐	☐	☐	☐
Ear punching	☐	☐	☐	☐	☐	☐	☐
Application per os with probe	☐	☐	☐	☐	☐	☐	☐
Application per os without probe	☐	☐	☐	☐	☐	☐	☐
Injection subcutaneous	☐	☐	☐	☐	☐	☐	☐
Injection intramuscular	☐	☐	☐	☐	☐	☐	☐
Injection intraperitoneal	☐	☐	☐	☐	☐	☐	☐
Injection intravenously dorsal penile vein (rat)	☐	☐	☐	☐	☐	☐	☐
Injection intravenous caudal vein mediana	☐	☐	☐	☐	☐	☐	☐
Blood sampling sublingual vein (rat)	☐	☐	☐	☐	☐	☐	☐
Blood collection facial vein (mouse)	☐	☐	☐	☐	☐	☐	☐
Blood sampling retrobulbar venous plexus	☐	☐	☐	☐	☐	☐	☐
Blood collection saphenous vein	☐	☐	☐	☐	☐	☐	☐
Blood sampling caudal vein mediana	☐	☐	☐	☐	☐	☐	☐
blood sampling intracardiac	☐	☐	☐	☐	☐	☐	☐
cervical dislocation (up to 100g)	☐	☐	☐	☐	☐	☐	☐
intubation	☐	☐	☐	☐	☐	☐	☐
vaginal smear	☐	☐	☐	☐	☐	☐	☐
microsurgical techniques	☐	☐	☐	☐	☐	☐	☐
suture techniques	☐	☐	☐	☐	☐	☐	☐
Other	☐	☐	☐	☐	☐	☐	☐

J4. (Condition J3: Select "other methods") Please briefly describe other methods.

J5. How well are the following anatomical features on Rat simulator A replicated?

Please assign school grades from 1 to 6:

1: very good; 2: good; 3: satisfactory; 4: sufficient; 5: poor; 6: insufficient.

	1	2	3	4	5	6	No
Haptics in general							
⇒ specifically: displaceability and consistency of the skin							
Body size							
Weight							
Body shape							
Proportions							
Presence of gender-specific Features							
Presence of blood vessels							
Location of the blood vessels							
Course of the blood vessels							
Condition of the blood vessels							
Presence of joints							
Mobility of the joints							

J6. How practical is Rat simulator A in the use of multiple courses?

Please indicate how well the following statements about Rat simulator A apply.

	Fully applies	Largely applies	Rather applies	Rather does not apply	Largely does not apply	Does not apply at all	No answer
The manual is detailed and easy to understand.	☐	☐	☐	☐	☐	☐	☐
The simulator is easy to store and transport.	☐	☐	☐	☐	☐	☐	☐
The simulator is easy to clean and disinfect.	☐	☐	☐	☐	☐	☐	☐
The simulator is made of disinfection-resistant material.	☐	☐	☐	☐	☐	☐	☐
The simulator is made of robust material.	☐	☐	☐	☐	☐	☐	☐
The simulator is made of realistic material.	☐	☐	☐	☐	☐	☐	☐
The acquisition costs are reasonable.	☐	☐	☐	☐	☐	☐	☐
The service life is reasonable.	☐	☐	☐	☐	☐	☐	☐
Running costs for spare parts and consumables are reasonable.	☐	☐	☐	☐	☐	☐	☐
Spare parts have a long service life.	☐	☐	☐	☐	☐	☐	☐
Spare parts are available for all trainable methods on the simulator.	☐	☐	☐	☐	☐	☐	☐
Spare parts are easy to replace.	☐	☐	☐	☐	☐	☐	☐

Part K: Rat simulator C (condition E1 selection "sporadic use" or "regular use" for Rat simulator C).

K1. How many years has the Rat simulator C been used in your course?

Please enter numbers only.

for about ☐☐☐☐ years

K2. How satisfied are you with Rat simulator C overall?

- very satisfied ☐
- Quite satisfied ☐
- Rather satisfied ☐
- rather dissatisfied ☐
- quite dissatisfied ☐
- Very dissatisfied ☐
- not specified ☐

K3. **How well can the following methods be practiced on Rat simulator C?**

Please assign school grades from 1 to 6: 1: very good; 2: good; 3: satisfactory; 4: sufficient; 5: poor; 6: insufficient; no training: method is not trained on the simulator.

	1	2	3	4	5	6	No
Handling	☐	☐	☐	☐	☐	☐	☐
Restraint	☐	☐	☐	☐	☐	☐	☐
Ear punching	☐	☐	☐	☐	☐	☐	☐
Application per os with probe	☐	☐	☐	☐	☐	☐	☐
Application per os without probe	☐	☐	☐	☐	☐	☐	☐
Injection subcutaneous	☐	☐	☐	☐	☐	☐	☐
Injection intramuscular	☐	☐	☐	☐	☐	☐	☐
Injection intraperitoneal	☐	☐	☐	☐	☐	☐	☐
Injection intravenously dorsal penile vein (rat)	☐	☐	☐	☐	☐	☐	☐
Injection intravenous caudal vein mediana	☐	☐	☐	☐	☐	☐	☐
Blood sampling sublingual vein (rat)	☐	☐	☐	☐	☐	☐	☐
Blood collection facial vein (mouse)	☐	☐	☐	☐	☐	☐	☐
Blood sampling retrobulbar venous plexus	☐	☐	☐	☐	☐	☐	☐
Blood collection saphenous vein	☐	☐	☐	☐	☐	☐	☐
Blood sampling caudal vein mediana	☐	☐	☐	☐	☐	☐	☐
blood sampling intracardiac	☐	☐	☐	☐	☐	☐	☐
cervical dislocation (up to 100g)	☐	☐	☐	☐	☐	☐	☐
intubation	☐	☐	☐	☐	☐	☐	☐
vaginal smear	☐	☐	☐	☐	☐	☐	☐
microsurgical techniques	☐	☐	☐	☐	☐	☐	☐
suture techniques	☐	☐	☐	☐	☐	☐	☐
Other	☐	☐	☐	☐	☐	☐	☐

K4. **(Condition K3: Select "other methods") Please briefly describe other methods.**

K5.b **How well are the following anatomical features formed on Rat simulator C?**

Please assign school grades from 1 to 6:

1: very good; 2: good; 3: satisfactory; 4: sufficient; 5: poor; 6: insufficient.

	1	2	3	4	5	6	No
Haptics in general	☐	☐	☐	☐	☐	☐	☐
⇒ specifically: displaceability and consistency if the skin	☐	☐	☐	☐	☐	☐	☐
Body size	☐	☐	☐	☐	☐	☐	☐
Weight	☐	☐	☐	☐	☐	☐	☐
Body shape	☐	☐	☐	☐	☐	☐	☐
Proportions	☐	☐	☐	☐	☐	☐	☐
Presence of gender-specific Features	☐	☐	☐	☐	☐	☐	☐
Presence of blood vessels	☐	☐	☐	☐	☐	☐	☐
Location of the blood vessels	☐	☐	☐	☐	☐	☐	☐
Course of the blood vessels	☐	☐	☐	☐	☐	☐	☐
Condition of the blood vessels	☐	☐	☐	☐	☐	☐	☐
Presence of joints	☐	☐	☐	☐	☐	☐	☐
Mobility of the joints	☐	☐	☐	☐	☐	☐	☐

K6. How practical is Rat simulator C in the use of multiple courses?

Please indicate how well the following statements about Rat simulator C apply.

	Fully applies	Largely applies	Rather applies	Rather does not apply	Largely does not apply	Does not apply at all	No answer
The manual is detailed and easy to understand.	☐	☐	☐	☐	☐	☐	☐
The simulator is easy to store and transport.	☐	☐	☐	☐	☐	☐	☐
The simulator is easy to clean and disinfect.	☐	☐	☐	☐	☐	☐	☐
The simulator is made of disinfection-resistant material.	☐	☐	☐	☐	☐	☐	☐
The simulator is made of robust material.	☐	☐	☐	☐	☐	☐	☐
The simulator is made of realistic material.	☐	☐	☐	☐	☐	☐	☐
The acquisition costs are reasonable.	☐	☐	☐	☐	☐	☐	☐
The service life is reasonable.	☐	☐	☐	☐	☐	☐	☐
Running costs for spare parts and consumables are reasonable.	☐	☐	☐	☐	☐	☐	☐
Spare parts have a long service life.	☐	☐	☐	☐	☐	☐	☐
Spare parts are available for all trainable methods on the simulator.	☐	☐	☐	☐	☐	☐	☐
Spare parts are easy to replace.	☐	☐	☐	☐	☐	☐	☐

Part L: Rat simulator D (condition E1 selection "sporadic use" or "regular use" for Rat simulator D)

L1. For how many years has Rat simulator D been used in your course?

Please enter numbers only.

for about ☐☐☐☐ years

L2. How satisfied are you with Rat simulator D overall?

- Very satisfied ☐
- Quite satisfied ☐
- Rather satisfied ☐
- rather dissatisfied ☐
- quite dissatisfied ☐
- Very dissatisfied ☐
- not specified ☐

L3. How well can the following methods be practiced on Rat simulator D?

Please assign school grades from 1 to 6: 1: very good; 2: good; 3: satisfactory; 4: sufficient; 5: poor; 6: insufficient; no training: method is not tainted on the simulator.

	1	2	3	4	5	6	No
Handling	☐	☐	☐	☐	☐	☐	☐
Restraint	☐	☐	☐	☐	☐	☐	☐
Ear punching	☐	☐	☐	☐	☐	☐	☐
Application per os with probe	☐	☐	☐	☐	☐	☐	☐
Application per os without probe	☐	☐	☐	☐	☐	☐	☐
Injection subcutaneous	☐	☐	☐	☐	☐	☐	☐
Injection intramuscular	☐	☐	☐	☐	☐	☐	☐
Injection intraperitoneal	☐	☐	☐	☐	☐	☐	☐
Injection intravenously dorsal penile vein (rat)	☐	☐	☐	☐	☐	☐	☐
Injection intravenous caudal vein mediana	☐	☐	☐	☐	☐	☐	☐
Blood sampling sublingual vein (rat)	☐	☐	☐	☐	☐	☐	☐
Blood collection facial vein (mouse)	☐	☐	☐	☐	☐	☐	☐
Blood sampling retrobulbar venous plexus	☐	☐	☐	☐	☐	☐	☐
Blood collection saphenous vein	☐	☐	☐	☐	☐	☐	☐
Blood sampling caudal vein mediana	☐	☐	☐	☐	☐	☐	☐
blood sampling intracardiac	☐	☐	☐	☐	☐	☐	☐
cervical dislocation (up to 100g)	☐	☐	☐	☐	☐	☐	☐
intubation	☐	☐	☐	☐	☐	☐	☐
vaginal smear	☐	☐	☐	☐	☐	☐	☐
microsurgical techniques	☐	☐	☐	☐	☐	☐	☐
suture techniques	☐	☐	☐	☐	☐	☐	☐
Other	☐	☐	☐	☐	☐	☐	☐

L4. **(Condition L3: Select "other methods") Please briefly describe other methods.**

L5. **How well are the following anatomical features on Rat simulator D replicated?**

Please assign school grades from 1 to 6:

1: very good; 2: good; 3: satisfactory; 4: sufficient; 5: poor; 6: insufficient.

	1	2	3	4	5	6	No
Haptics in general	☐	☐	☐	☐	☐	☐	☐
⇒ specifically: displaceability and consistency of the skin	☐	☐	☐	☐	☐	☐	☐
Body size	☐	☐	☐	☐	☐	☐	☐
Weight	☐	☐	☐	☐	☐	☐	☐
Body shape	☐	☐	☐	☐	☐	☐	☐
Proportions	☐	☐	☐	☐	☐	☐	☐
Presence of gender-specific Features	☐	☐	☐	☐	☐	☐	☐
Presence of blood vessels	☐	☐	☐	☐	☐	☐	☐
Location of the blood vessels	☐	☐	☐	☐	☐	☐	☐
Course of the blood vessels	☐	☐	☐	☐	☐	☐	☐
Condition of the blood vessels	☐	☐	☐	☐	☐	☐	☐
Presence of joints	☐	☐	☐	☐	☐	☐	☐
Mobility of the joints	☐	☐	☐	☐	☐	☐	☐

L6. How practical is Rat simulator D in the use of multiple courses?

Please indicate how true the following statements are about Rat simulator D.

	Fully applies	Largely applies	Rather applies	Rather does not apply	Largely does not apply	Does not apply at all	No answer
The manual is detailed and easy to understand.	☐	☐	☐	☐	☐	☐	☐
The simulator is easy to store and transport.	☐	☐	☐	☐	☐	☐	☐
The simulator is easy to clean and disinfect.	☐	☐	☐	☐	☐	☐	☐
The simulator is made of disinfection-resistant material.	☐	☐	☐	☐	☐	☐	☐
The simulator is made of robust material.	☐	☐	☐	☐	☐	☐	☐
The simulator is made of realistic material.	☐	☐	☐	☐	☐	☐	☐
The acquisition costs are reasonable.	☐	☐	☐	☐	☐	☐	☐
The service life is reasonable.	☐	☐	☐	☐	☐	☐	☐
Running costs for spare parts and consumables are reasonable.	☐	☐	☐	☐	☐	☐	☐
Spare parts have a long service life.	☐	☐	☐	☐	☐	☐	☐
Spare parts are available for all trainable methods on the simulator.	☐	☐	☐	☐	☐	☐	☐
Spare parts are easy to replace.	☐	☐	☐	☐	☐	☐	☐

Part M: Rat simulator E Condition E1 Selection "sporadic use " or "regular use" for Rat simulator E).

M1. For how many years has Rat simulator E been used in your course?

Please enter numbers only.

for about ☐☐☐☐ years

M2. How satisfied are you with Rat simulator E overall?

- very satisfied ☐
- Quite satisfied ☐
- Rather satisfied ☐
- rather dissatisfied ☐
- quite dissatisfied ☐
- Very dissatisfied ☐
- not specified ☐

M3. How well can the following methods be practiced on Rat simulator E?

Please assign school grades from 1 to 6: 1: very good; 2: good; 3: satisfactory; 4: sufficient; 5: poor; 6: insufficient; no training: method is not trained on the simulator.

	1	2	3	4	5	6	No
Handling	☐	☐	☐	☐	☐	☐	☐
Restrain	☐	☐	☐	☐	☐	☐	☐
Ear punching	☐	☐	☐	☐	☐	☐	☐
Application per os with probe	☐	☐	☐	☐	☐	☐	☐
Application per os without probe	☐	☐	☐	☐	☐	☐	☐
Injection subcutaneous	☐	☐	☐	☐	☐	☐	☐
Injection intramuscular	☐	☐	☐	☐	☐	☐	☐
Injection intraperitoneal	☐	☐	☐	☐	☐	☐	☐
Injection intravenously dorsal penile vein (rat)	☐	☐	☐	☐	☐	☐	☐
Injection intravenous caudal vein mediana	☐	☐	☐	☐	☐	☐	☐
Blood sampling sublingual vein (rat)	☐	☐	☐	☐	☐	☐	☐
Blood collection facial vein (mouse)	☐	☐	☐	☐	☐	☐	☐
Blood sampling retrobulbar venous plexus	☐	☐	☐	☐	☐	☐	☐
Blood collection saphenous vein	☐	☐	☐	☐	☐	☐	☐
Blood sampling caudal vein mediana	☐	☐	☐	☐	☐	☐	☐
blood sampling intracardiac	☐	☐	☐	☐	☐	☐	☐
cervical dislocation (up to 100g)	☐	☐	☐	☐	☐	☐	☐
intubation	☐	☐	☐	☐	☐	☐	☐
vaginal smear	☐	☐	☐	☐	☐	☐	☐
microsurgical techniques	☐	☐	☐	☐	☐	☐	☐
suture techniques	☐	☐	☐	☐	☐	☐	☐
Other	☐	☐	☐	☐	☐	☐	☐

M4. **(Condition M3: Selection "other methods) Please describe other methods briefly.**

M5. How well are the following anatomical features on Rat simulator E replicated?

Please assign school grades from 1 to 6:

1: very good; 2: good; 3: satisfactory; 4: sufficient; 5: poor; 6: insufficient.

	1	2	3	4	5	6	No
Haptics in general	☐	☐	☐	☐	☐	☐	☐
⇒ specifically: displaceability and consistency of the skin	☐	☐	☐	☐	☐	☐	☐
Body size	☐	☐	☐	☐	☐	☐	☐
Weight	☐	☐	☐	☐	☐	☐	☐
Body shape	☐	☐	☐	☐	☐	☐	☐
Proportions	☐	☐	☐	☐	☐	☐	☐
Presence of gender-specific Features	☐	☐	☐	☐	☐	☐	☐
Presence of blood vessels	☐	☐	☐	☐	☐	☐	☐
Location of the blood vessels	☐	☐	☐	☐	☐	☐	☐
Course of the blood vessels	☐	☐	☐	☐	☐	☐	☐
Condition of the blood vessels	☐	☐	☐	☐	☐	☐	☐
Presence of joints	☐	☐	☐	☐	☐	☐	☐
Mobility of the joints	☐	☐	☐	☐	☐	☐	☐

M6. How practical is Rat simulator E in the use of multiple courses?

Please indicate how well the following statements about Rat simulator E apply.

	Fully applies	Largely applies	Rather applies	Rather does not apply	Largely does not apply	Does not apply at all	No answer
The manual is detailed and easy to understand.	☐	☐	☐	☐	☐	☐	☐
The simulator is easy to store and transport.	☐	☐	☐	☐	☐	☐	☐
The simulator is easy to clean and disinfect.	☐	☐	☐	☐	☐	☐	☐
The simulator is made of disinfection-resistant material.	☐	☐	☐	☐	☐	☐	☐
The simulator is made of robust material.	☐	☐	☐	☐	☐	☐	☐
The simulator is made of realistic material.	☐	☐	☐	☐	☐	☐	☐
The acquisition costs are reasonable.	☐	☐	☐	☐	☐	☐	☐
The service life is reasonable.	☐	☐	☐	☐	☐	☐	☐
Running costs for spare parts and consumables are reasonable.	☐	☐	☐	☐	☐	☐	☐
Spare parts have a long service life.	☐	☐	☐	☐	☐	☐	☐
Spare parts are available for all trainable methods on the simulator.	☐	☐	☐	☐	☐	☐	☐
Spare parts are easy to replace.	☐	☐	☐	☐	☐	☐	☐

Part N: Mouse simulator (condition E1 selection "sporadic use " or "regular use" for mouse simulator).

N1. How many years has Mouse simulator been used in your

course?

Please enter numbers only.

for about ☐☐☐☐ years

N2. How satisfied are you with Mouse simulator overall?

- very satisfied ☐
- Quite satisfied ☐
- Rather satisfied ☐
- rather dissatisfied ☐
- quite dissatisfied ☐
- Very dissatisfied ☐
- not specified ☐

N3. How well can the following methods be practiced on Mouse simulator?

Please assign school grades from 1 to 6: 1: very good; 2: good; 3: satisfactory; 4: sufficient; 5: poor; 6: insufficient; no training: method is not trained on the simulator.

	1	2	3	4	5	6	No
Handling	☐	☐	☐	☐	☐	☐	☐
Restraint	☐	☐	☐	☐	☐	☐	☐
Ear punching	☐	☐	☐	☐	☐	☐	☐
Application per os with probe	☐	☐	☐	☐	☐	☐	☐
Application per os without probe	☐	☐	☐	☐	☐	☐	☐
Injection subcutaneous	☐	☐	☐	☐	☐	☐	☐
Injection intramuscular	☐	☐	☐	☐	☐	☐	☐
Injection intraperitoneal	☐	☐	☐	☐	☐	☐	☐
Injection intravenously dorsal penile vein (rat)	☐	☐	☐	☐	☐	☐	☐
Injection intravenous caudal vein mediana	☐	☐	☐	☐	☐	☐	☐
Blood sampling sublingual vein (rat)	☐	☐	☐	☐	☐	☐	☐
Blood collection facial vein (mouse)	☐	☐	☐	☐	☐	☐	☐
Blood sampling retrobulbar venous plexus	☐	☐	☐	☐	☐	☐	☐
Blood collection saphenous vein	☐	☐	☐	☐	☐	☐	☐
Blood sampling caudal vein mediana	☐	☐	☐	☐	☐	☐	☐
blood sampling intracardiac	☐	☐	☐	☐	☐	☐	☐
cervical dislocation (up to 100g)	☐	☐	☐	☐	☐	☐	☐
intubation	☐	☐	☐	☐	☐	☐	☐
vaginal smear	☐	☐	☐	☐	☐	☐	☐
microsurgical techniques	☐	☐	☐	☐	☐	☐	☐
suture techniques	☐	☐	☐	☐	☐	☐	☐
Other		☐	☐	☐	☐	☐	☐

N4. (Condition N3: Select "other methods") Please briefly describe other methods.

N5. How good are the following anatomical features on Mouse simulator replicated?

Please assign school grades from 1 to 6:

1: very good; 2: good; 3: satisfactory; 4: sufficient; 5: poor; 6: insufficient.

	1	2	3	4	5	6	No
Haptics in general	☐	☐	☐	☐	☐	☐	☐
⇒ speci ically: displaceability and consistency of the skin skin	☐	☐	☐	☐	☐	☐	☐
Body size	☐	☐	☐	☐	☐	☐	☐
Weight	☐	☐	☐	☐	☐	☐	☐
Body shape	☐	☐	☐	☐	☐	☐	☐
Proportions	☐	☐	☐	☐	☐	☐	☐
Presence of gender-specific Features	☐	☐	☐	☐	☐	☐	☐
Presence of blood vessels	☐	☐	☐	☐	☐	☐	☐
Location of the blood vessels	☐	☐	☐	☐	☐	☐	☐
Course of the blood vessels	☐	☐	☐	☐	☐	☐	☐
Condition of the blood vessels	☐	☐	☐	☐	☐	☐	☐
Presence of joints	☐	☐	☐	☐	☐	☐	☐
Mobility of the joints	☐	☐	☐	☐	☐	☐	☐

N6. How practical is Mouse simulator in the use of multiple courses?

Please indicate how true the following statements are about Mouse simulator.

	Fully applies	Largely applies	Rather applies	Rather does not apply	Largely does not apply	Does not apply at all	No answer
The manual is detailed and easy to understand.	☐	☐	☐	☐	☐	☐	☐
The simulator is easy to store and transport.	☐	☐	☐	☐	☐	☐	☐
The simulator is easy to clean and disinfect.	☐	☐	☐	☐	☐	☐	☐
The simulator is made of disinfection-resistant material.	☐	☐	☐	☐	☐	☐	☐
The simulator is made of robust material.	☐	☐	☐	☐	☐	☐	☐
The simulator is made of realistic material.	☐	☐	☐	☐	☐	☐	☐
The acquisition costs are reasonable.	☐	☐	☐	☐	☐	☐	☐
The service life is reasonable.	☐	☐	☐	☐	☐	☐	☐
Running costs for spare parts and consumables are reasonable.	☐	☐	☐	☐	☐	☐	☐
Spare parts have a long service life.	☐	☐	☐	☐	☐	☐	☐
Spare parts are available for all trainable methods on the simulator.	☐	☐	☐	☐	☐	☐	☐
Spare parts are easy to replace.	☐	☐	☐	☐	☐	☐	☐

Part O: Rat simulator F (condition E1 selection "sporadic use " or "regular use" for Rat simulator F).

O1. How many years has Rat simulator F been used in your course?

Please enter numbers only.

since about [ ] Years

O2. How satisfied are you with Rat simulator F overall?

- very satisfied ☐
- fairly satisfied ☐
- rather satisfied ☐
- rather dissatisfied ☐
- fairly dissatisfied ☐
- very dissatisfied ☐
- not specified ☐

O3. How well can the following methods be practiced on Rat simulator F?

Please assign school grades from 1 to 6:

1: very good; 2: good; 3: satisfactory; 4: sufficient; 5: poor; 6: insufficient; no training: method is not trained on the simulator.

	1	2	3	4	5	6	No training
microsurgical techniques	☐	☐	☐	☐	☐	☐	☐
Suture	☐	☐	☐	☐	☐	☐	☐
techniques other	☐	☐	☐	☐	☐	☐	☐

O4. b (Condition O3: Selection "other methods") Please briefly describe other methods.

O5. How well are the following anatomical features on Rat simulator F replicated?

Please assign school grades from 1 to 6:

1: very good; 2: good; 3: satisfactory; 4: sufficient; 5: poor; 6: insufficient.

	1	2	3	4	5	6	
Haptics in general	☐	☐	☐	☐	☐	☐	☐
⇒ special: consistency of the skin	☐	☐	☐	☐	☐	☐	☐
Body size	☐	☐	☐	☐	☐	☐	☐
Body shape	☐	☐	☐	☐	☐	☐	☐
Proportions	☐	☐	☐	☐	☐	☐	☐
Presence of gender-specific Features	☐	☐	☐	☐	☐	☐	☐
Presence of blood vessels	☐	☐	☐	☐	☐	☐	☐
Location of the blood vessels	☐	☐	☐	☐	☐	☐	☐
Course of the blood vessels	☐	☐	☐	☐	☐	☐	☐
Condition of the blood vessels	☐	☐	☐	☐	☐	☐	☐
Position of the kidney	☐	☐	☐	☐	☐	☐	☐
Nature of the kidney	☐	☐	☐	☐	☐	☐	☐
Kidney shape	☐	☐	☐	☐	☐	☐	☐
Location of the bladder	☐	☐	☐	☐	☐	☐	☐
Condition of the bladder	☐	☐	☐	☐	☐	☐	☐
Bladder shape	☐	☐	☐	☐	☐	☐	☐
Presence of the trachea	☐	☐	☐	☐	☐	☐	☐
Location of the trachea	☐	☐	☐	☐	☐	☐	☐
Condition of the trachea	☐	☐	☐	☐	☐	☐	☐
Presence of the esophagus	☐	☐	☐	☐	☐	☐	☐
Location of the esophagus	☐	☐	☐	☐	☐	☐	☐
The nature of the esophagus	☐	☐	☐	☐	☐	☐	☐

O6. How practical is Rat simulator F in the use of multiple courses?

Please indicate how well the following statements about Rat simulator F apply.

	Fully applies	Largely applies	Rather applies	Rather does not apply	Largely does not apply	Does not apply at all	No answer
The manual is detailed and easy to understand.	☐	☐	☐	☐	☐	☐	☐
The simulator is easy to store and transport.	☐	☐	☐	☐	☐	☐	☐
The simulator is easy to clean and disinfect.	☐	☐	☐	☐	☐	☐	☐
The simulator is made of disinfection-resistant material.	☐	☐	☐	☐	☐	☐	☐
The simulator is made of robust material.	☐	☐	☐	☐	☐	☐	☐
The simulator is made of realistic material.	☐	☐	☐	☐	☐	☐	☐
The acquisition costs are reasonable.	☐	☐	☐	☐	☐	☐	☐
The service life is reasonable.	☐	☐	☐	☐	☐	☐	☐
Running costs for spare parts and consumables are reasonable.	☐	☐	☐	☐	☐	☐	☐
Spare parts have a long service life.	☐	☐	☐	☐	☐	☐	☐
Spare parts are available for all trainable methods on the simulator.	☐	☐	☐	☐	☐	☐	☐
Spare parts are easy to replace.	☐	☐	☐	☐	☐	☐	☐

.

.

Part P: Methodological requirements for a new simulator

The last part of the survey is about your requirements for a new simulator. The questions are divided into methodological, anatomical and practical requirements.

1. Methodological requirements for a new simulator

⇒ Which methods are important to you?

P1. Which methods would you like to train on a new rat simulator?

Arrange a maximum of 10 methods in the right list (highest priority on top). A double click moves an element to the other list. The elements can be moved with the mouse.

Handling	☐☐
Restraint	☐☐
Ear punch	☐☐
Application per os by via feeding tube	☐☐
Application per os without tube	☐☐
Injection subcutaneous	☐☐
Injection intramuscular	☐☐
Injection intraperitoneal	☐☐
Injection intravenously dorsal penile vein	☐☐
Injection intravenous caudal vein mediana	☐☐
Blood sampling sublingual vein	☐☐
Blood sampling retrobulbar venous plexus	☐☐
Blood sampling saphenous vein	☐☐
Blood sampling caudal vein mediana	☐☐
Blood sampling intracardial	☐☐
cervical dislocation (up to 100g)	☐☐
Intubation	☐☐
Vaginal smear	☐☐
Suture techniques	☐☐
other methods	☐☐

P2. (Condition P1 selection "Other methods") What other methods would you like to use fpr practice on a rat simulator?

P3. Which methods would you like to train on a new mouse simulator?

Arrange a maximum of 10 methods in the right list (highest priority at the top).
A double click moves an element to the other list. The elements can be moved with the mouse.

- Handling
- Restraint
- Ear punch
- Application per os via feeding tube
- Application per os without tube
- Injection subcutaneous
- Injection intramuscular
- Injection intraperitoneal
- Injection intravenous caudal vein mediana
- Blood sampling facial vein
- Blood sampling retrobulbar venous plexus
- Blood sampling saphenous vein
- Blood sampling caudal vein mediana
- Blood sampling intracardial
- cervical dislocation (up to 100g)
- Intubation
- Vaginal smear
- microsurgical techniques
- Suture techniques
- Other methods

P4. (Condition P3: Selection "other methods")
What other methods would you like to train on the mouse simulator?

Part Q: Anatomical requirements for a new simulator

2. Anatomical requirements for a new simulator

⇒ In your opinion, which structures must be reproduced as faithfully as possible?

Q1. **How important is it to you to reproduce anatomical conditions as correctly as possible overall?**

- very important ☐
- quite important ☐
- rather important ☐
- rather unimportant ☐
- quite unimportant ☐
- very unimportant ☐
- not specified ☐

Q2. How important is it to you to reproduce general anatomical features as correctly as possible?

	very import ant	quite import ant	rathe r import ant	rather unimport ant	quite unimport ant	very unimport ant	not specifie d
Haptics in general	☐	☐	☐	☐	☐	☐	☐
⇒ specifically: displaceability and consistency of the skin	☐	☐	☐	☐	☐	☐	☐
Body size	☐	☐	☐	☐	☐	☐	☐
Weight	☐	☐	☐	☐	☐	☐	☐
Body shape	☐	☐	☐	☐	☐	☐	☐
Proportions	☐	☐	☐	☐	☐	☐	☐
Presence of gender-specific Features	☐	☐	☐	☐	☐	☐	☐
Presence of blood vessels	☐	☐	☐	☐	☐	☐	☐
Location of the blood vessels	☐	☐	☐	☐	☐	☐	☐
Course of the blood vessels	☐	☐	☐	☐	☐	☐	☐
Condition of the blood vessels	☐	☐	☐	☐	☐	☐	☐
Presence of joints	☐	☐	☐	☐	☐	☐	☐
Mobility of the joints	☐	☐	☐	☐	☐	☐	☐

Q3.How important is it to them to replicate the head and neck region as correctly as possible?

	very important	quite important	rather important	rather unimportant	quite unimportant	very unimportant	not specified
Sensory organs in general	☐	☐	☐	☐	☐	☐	☐
⇒ especially eyes	☐	☐	☐	☐	☐	☐	☐
⇒ especially ears	☐	☐	☐	☐	☐	☐	☐
⇒ especially nose	☐	☐	☐	☐	☐	☐	☐
⇒ especially whiskers	☐	☐	☐	☐	☐	☐	☐
⇒ especially skin	☐	☐	☐	☐	☐	☐	☐
Organs of the oral cavity in general	☐	☐	☐	☐	☐	☐	☐
⇒ specially teeth	☐	☐	☐	☐	☐	☐	☐
⇒ especially tongue	☐	☐	☐	☐	☐	☐	☐
⇒ especially temporomandibular joint	☐	☐	☐	☐	☐	☐	☐
⇒ especially larynx	☐	☐	☐	☐	☐	☐	☐
Presence of the trachea	☐	☐	☐	☐	☐	☐	☐
Location of the trachea	☐	☐	☐	☐	☐	☐	☐
Condition of the trachea	☐	☐	☐	☐	☐	☐	☐
Presence of the esophagus	☐	☐	☐	☐	☐	☐	☐
Location of the esophagus	☐	☐	☐	☐	☐	☐	☐
The nature of the esophagus	☐	☐	☐	☐	☐	☐	☐
other characteristic / other characteristics	☐	☐	☐	☐	☐	☐	☐

Q4. (Condition Q3 selection "other feature(s)") Which other feature of the head and neck region is important to you?

Q5. (Condition Q3 selection "other characteristic(s)") Which other feature of the head and neck region is unimportant to you?

Part R: Anatomical requirements for a new simulator

2. Anatomical requirements for a new simulator

⇒Which structures are important to you?

R1. **How important is the presence of the following replicas of the sensory organs to you?**

Please rank the sense organs according to their importance (highest priority on top). If a sense organ is unimportant, please leave it on the left side. A double click moves an element to the other side. The elements can be moved with the mouse.

Eyes ☐

Ears ☐

Nose ☐

Whiskers ☐

Skin ☐

R2. **How important to you is the presence of the following replicas of the organs of the oral cavity and neck?**

Please rank the organs according to their importance (highest priorityb on top). If an organ is unimportant, please leave it on the left side. A double click moves an element to the other side. The elements can be moved with the mouse.

Teeth ☐

Tongue ☐

TMJ (temporomandibular joint) ☐

Larynx ☐

Esophagus ☐

Trachea ☐

Other organ ☐

R3. (Condition R2: Selection "other organ")

What other organ of the oral cavity and neck are you interested in replicating?

R4. **How important to you is the presence of the following blood vessels in a rat simulator?**

Please order the blood vessels according to their importance (highest priority on top). If a blood vessel is unimportant, please leave it on the left side. A double click moves an element to the other side. The elements can be moved with the mouse.

Sublingual vein ☐

retrobulbar venous plexus ☐

Saphenous vein ☐

Vena caudalis mediana ☐

Other vessel ☐

R5. (Condition R4: Selection "other vessel")
What other blood vessel is important to you in the rat simulator?

R6.How important to you is the presence of the following blood vessels in a mouse simulator?

Please order the blood vessels according to their importance (highest priority on top). If a blood vessel is unimportant, please leave it on the left side. A double click moves an element to the other side. The elements can be moved with the mouse.

Facial vein ☐

retrobulbar venous plexus ☐

Saphenous vein ☐

Vena caudalis mediana ☐

Other vessel ☐

R7. (Condition R6: Selection "other vessel")
What other blood vessel is important to you in the mouse simulator?

Part S: Practical requirements for a new simulator

3. Practical requirements for a new simulator

⇒ What features are important to you when used in multiple courses?

S1. How important are the following features to you when used in multiple courses?

	very important	quite important	rather important	rather unimport-ant	quite unimport-ant	very unimport-ant	not specified
The manual is detailed and easy to understand.	☐	☐	☐	☐	☐	☐	☐
The simulator is easy to store and transport.	☐	☐	☐	☐	☐	☐	☐
The simulator is easy to clean and disinfect.	☐	☐	☐	☐	☐	☐	☐
The simulator is made of disinfection-resistant material.	☐	☐	☐	☐	☐	☐	☐
The simulator is made of robust material.	☐	☐	☐	☐	☐	☐	☐
The simulator is made of realistic material.	☐	☐	☐	☐	☐	☐	☐
The acquisition costs are reasonable.	☐	☐	☐	☐	☐	☐	☐
The service life is reasonable.	☐	☐	☐	☐	☐	☐	☐
Running costs for spare parts and consumables are reasonable.	☐	☐	☐	☐	☐	☐	☐
Spare parts have a long service life.	☐	☐	☐	☐	☐	☐	☐
Spare parts are available for all trainable methods on the simulator.	☐	☐	☐	☐	☐	☐	☐
Spare parts are easy to replace.	☐	☐	☐	☐	☐	☐	☐

Part T: Further requirements for a new simulator

Are any other features important to you? We look forward to receiving your information!

T1. What other criteria are important to you for a new development?

methodological requirements

anatomical requirements

practical requirements

Part U: Questions about yourself

U1. How old are you?

U2. What gender are you?

female

male

diverse

not specified

U3. How long have you been conducting experimental animal science courses for rat and mouse?

U4. Are you a course trainer or course supervisor?

I am a course trainer

I am a course supervisor.

not specified

Other

Other

U5. Comments, praise & criticism?

Here you can leave me your message.

We sincerely thank you for your support!

We would like to draw your attention to the second part of our evaluation: The Evaluation in Courses - An Assessment of Simulators from the Course Participants' Perspective. If you are interested in participating in the evaluation from the course participants' perspective with your courses or if you would like further information, you can leave a contact via the link https://survey.vetmed.fu-berlin.de/index.php/455897?lang=en or write to us personally via *kontakt@simulRATor.de.* We would also be happy to send you a summary of the study results by e-mail upon request. To do so, please write to us at *kontakt@simulRATor.de.* *PRIVACY NOTICE: Your contact information will NOT be associated with the answers to the questions.

***Please use kontakt@simulRATor.de for any* questions. We are looking forward to your message! Thank you very much for your support!**

***Melanie Humpenöder melanie.humpenoeder@fu-berlin.de* Institute for Animal Welfare, Behavior and Laboratory Animal Science Königsweg 67 14163 Berlin *Giuliano Mario Corte giuliano.corte@fu-berlin.de* Institute for Veterinary Anatomy Koserstraße 20 14059 Berlin Institute for Veterinary Epidemiology and Biometry Königsweg 67 14163 Berlin**
